# Design, Synthesis, Molecular Docking, and ADME-Tox Investigations of Imidazo[1,2-a]Pyrimidines Derivatives as Antimicrobial Agents

**DOI:** 10.3390/molecules29215058

**Published:** 2024-10-26

**Authors:** Djamila Benzenine, Ismail Daoud, Nadia Aissaoui, Zahira Kibou, Julio A. Seijas, M. Pilar Vázquez-Tato, Chewki Ziani-Cherif, Lahcen Belarbi, Noureddine Choukchou-Braham

**Affiliations:** 1 Laboratoire de Catalyse et Synthèse en Chimie Organique, Faculté des Sciences, Université de Tlemcen, BP 119, Tlemcen 13000, Algeria; djamila.benzenine@univ-temouchent.edu.dz (D.B.);; 2 Faculté des Sciences et de la Technologie, Université de Ain Témouchent, BP 284, Ain Témouchent 46000, Algeria; 3 Department of Matter Sciences, University Mohamed Khider, BP 145 RP, Biskra 07000, Algeria; i.daoud@univ-biskra.dz; 4 Laboratory of Natural and Bioactive Substances, University of Abou-Bakr Belkaid, BP 119, Tlemcen 13000, Algeria; 5 Laboratory of the Sustainable Management of Natural Resources in Arid and Semi-Arid Areas, Institute of Sciences, University Center of Naama, BP 66, Naama 45000, Algeria; aissaoui.nadia@cuniv-naama.dz; 6 Departamento de Química Orgánica, Facultad de Ciencias, Universidad de Santiago de Compostela, Avda. Alfonso X El Sabio s/n, 27002 Lugo, Spain; pilar.vazquez.tato@usc.es; 7 Laboratorie de Chimie Appliquée, Université de Ain Témouchent, BP 284, Ain Témouchent 46000, Algeria

**Keywords:** imidazo[1,2-*a*]pyrimidine, microwave, Al_2_O_3_ catalyst, solvent free, antimicrobial activity, in silico study

## Abstract

A convenient and effective synthesis of imidazo[1,2-*a*]pyrimidine derivatives has been developed under microwave irradiations using Al_2_O_3_ as a catalyst in solvent-free conditions. The functionalized imidazo[1,2-*a*]pyrimidine derivatives are useful in biochemistry and medical science. In our investigation, the antimicrobial activity of the synthesized compounds was evaluated against 13 microorganisms, including 6 Gram-positive bacteria, 4 Gram-negative bacteria, and 3 pathogenic fungi. Bioactivity tests revealed that the majority of the compounds exhibited good antimicrobial activity. Finally, molecular docking simulations and ADME-T predictions were performed, showing that the most active compounds have good binding modes with microbial targets and promising pharmacokinetic safety profiles.

## 1. Introduction

**Figure 1:**
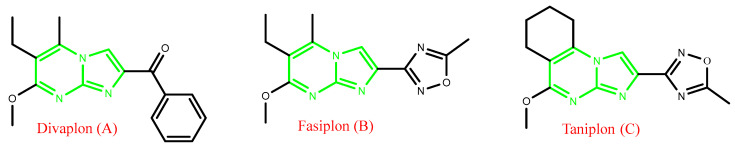
Pharmacologically active drugs holding imidazo[1,2-*a*]pyrimidine scaffolds.

Aza-heterocycles have found their outstanding place among bioactive products due to their important functions in the molecular forms of life. They are considered as an essential type of bio-organic molecules playing a particular role among a variety of significant natural and medicinal products [1,2]. As one of the aromatic heterocyclic rings and imidazopyrimidines, they show pharmaceutical properties of interest because of their important therapeutic activities [3]. Imidazo[1,2-*a*]pyrimidines are a heterocyclic scaffold of great interest because they exhibit a variety of physiological properties, such as antioxidant [4], anti-inflammatory [5], anxiolytic [6], anticonvulsant [7], benzodiazepine receptor agonist [8], calcium channel blocker [9], antitubercular [10], anticancer [11], antimalarial [12], antiviral [13], antimicrobial [14], and antifungal effects [15]. Moreover, imidazo[1,2-*a*]pyrimidines are found in various drugs as a basic unit of commercially accessible compound libraries, for example, divaplon, fasiplon, and taniplon (Figure 1) [16]. This is possibly a result of their exclusive physicochemical characteristics and their similarity to natural substrates like purines [17]. Imidazo[1,2-*a*]pyrimidine heterocycles exhibit significant biological activities. Their structural analogy to purine heterocycles and the guanidine functionality suggest multiple possible biological activities. Nevertheless, a deeper investigation is important to fully evaluate the biological effectiveness and range of this bicyclic framework, particularly in the area of antimicrobial research [14].

Antimicrobial resistance occurs when viruses, bacteria, and fungi do not respond effectively to drugs anymore. It complicates the treatment of infection, increases the risk of disease transmission, and may even cause death [18]. It is not surprising, therefore, that considerable efforts have been directed toward developing synthetic approaches for constructing various antimicrobial compounds. As a part of our continuous works [19,20,21,22,23,24,25], we herein aim to report a new synthesis of imidazo[1,2-*a*]pyrimidine derivatives from 2-aminopyrimidine and *α*-bromoketones. The reaction was activated with an aluminum-based catalyst, irradiated by a domestic microwave under solvent-free conditions, marking the first synthesis of 2-arylimidazo[1,2-*a*]pyrimidines utilizing Al_2_O_3_ as a catalyst. To the best of our knowledge, compounds **3e** and **3k** are novel derivatives of the imidazo[1,2-a]pyrimidine family, characterized by the presence of a methoxy group in the *ortho* position and two methyl groups at the *meta* and *para* positions of the benzene ring, respectively. The antimicrobial activity of synthesized heterocyclic compounds was examined by in vitro and in silico studies.

The design, manufacturing, and synthesis of compounds valuable as human therapeutic molecules are one of the most important goals of medicinal chemistry and organic synthesis [26,27]. For this purpose, and to study their antimicrobial inhibitory activities, eleven imidazo[1,2-*a*]pyrimidines were synthesized under microwave irradiation catalyzed by an aluminum-based catalyst. The in vitro antimicrobial inhibitory activities of all prepared heterocyclic compounds were evaluated. Moreover, the inhibitory mechanism of some imidazo[1,2-*a*]pyrimidine derivatives was investigated through molecular docking and enzyme kinetic studies. Our study can provide lead molecules for the search and expansion of natural compounds based on antimicrobial candidates.

## 2. Results and Discussion

### 2.1. Chemistry

**Scheme 1:**
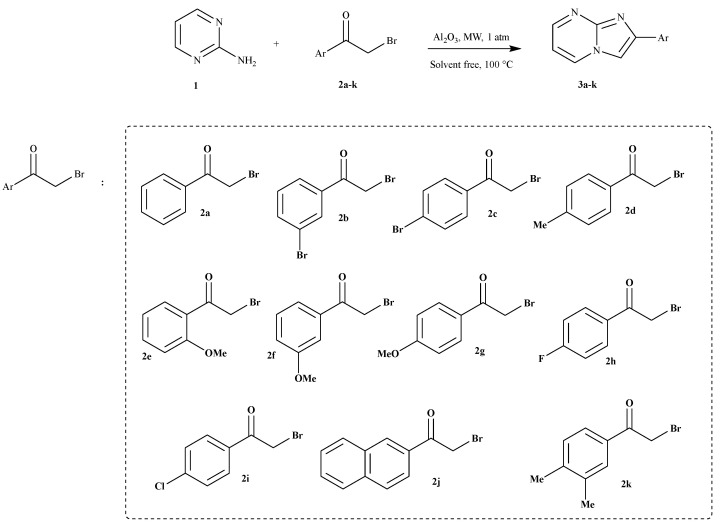
Synthesis of imidazo[1,2-*a*]pyrimidine derivatives **3a**–**k.**

As shown in Scheme 1, imidazo[1,2-*a*]pyrimidines **3a**–**k** were synthesized by a condensation reaction of 2-aminopyrimidine and several 2-bromoarylketones without solvent. They were catalyzed by basic alumina (Al_2_O_3_) and irradiated using a domestic microwave.

To optimize the synthesis of 2-(4-chlorophenyl)imidazo[1,2-*a*]pyrimidine, the amount of catalyst was varied to assess its impact on reaction efficiency. Table 1 summarizes the results of this optimization, highlighting the relationship between the amount of Al_2_O_3_ and the corresponding yields.

With these results in hand, the scope of the reaction was then studied (Table 2). This protocol was found suitable for a range of 2-bromoketones bearing electron-rich (Me or MeO) or electron-withdrawing groups (F or Br) on the *ortho*, *para*, or *meta* position of the benzene ring and proceeded smoothly, affording the corresponding products in 52–68% yields. It should be noted that unsubstituted 2-phenylimidazo[1,2-*a*]pyrimidine and the 2-naphtylimidazo[1,2-*a*]pyrimidine were also suitable for this transformation, and satisfactory yields (70 and 67%) were, respectively, obtained. The 3,4-dimethyl-substituted substrate worked well and provided a product with a 64% yield. In this study, we found that basic Al_2_O_3_ is an environmentally friendly catalytic system for the synthesis of imidazo[1,2-*a*]pyrimidine derivatives with good catalytic efficiency.

Despite the advantages of the use of Al_2_O_3_ as a catalyst like low toxicity, high surface area, and thermal stability, it led to lower-than-expected yields in this synthesis. Nonetheless, it is essential to evaluate this result in the context of green chemistry. Al_2_O_3_ serves as a non-toxic, abundant, and sustainable catalyst that reduces environmental harm. While the yields were lower than our expectations, our results highlight the prospective avenues for further optimization of reaction conditions and the investigation of hybrid catalytic systems that could enhance yields while adhering to green practices.

### 2.2. Biology

The results of the antimicrobial potency of **3c**–**k** are listed in Table 3. They show that compounds **3a**, **3c**, **3j**, **3g**, and **3k** exhibited strong activity against *C. albicans* species with an inhibitory zone ranging from 12.3 ± 0.5 mm to 19.3 ± 0.3 mm (see Supplementary Materials). Even in the case where compounds **3c**, **3g**, and **3j** displayed moderate activity against *S. aureus* and *M. luteus,* compound **3g** exhibited excellent activity against *B. subtilis* (19.3 ± 0.3 mm). However, none of the compounds revealed any activity against Gram-negative bacteria, *L. monocytogenes* and *E. faecalis*. In general, Gram-positive bacteria are more sensitive than Gram-negative ones. This sensitivity might be related to the cell envelope’s composition. The Gram-positive wall is formed by a thick layer of peptidoglycan, which facilitates the penetration of active agents in the action site [29]. From a structural point of view, it can be concluded that Br, OMe, Cl, and F in the *para* direction exhibited excellent antimicrobial potency; however, other substituents revealed quenching activity.

The antimicrobial potency of the active compounds that have an inhibitory zone was equal to or greater than 12 mm; these compounds were assessed against the sensitive microorganisms to determine their minimum inhibitory concentration (MIC).

Ten serial dilutions were prepared from an initial concentration of 80 mg/mL by reducing the stock solutions twofold. The results of the active compounds are summarized in Table 4. The results revealed varied activities among the active compounds, with MIC values ranging from 20 mg/mL to 2.5 mg/mL (Table 4). Compounds **3j** and **3k** exhibited excellent activities against *C. albicans*, while **3g** demonstrated activity against *B. subtilis* and *C. albicans*.

The MIC values were equal to MBC values in the most active imidazo[1,2-*a*]pyrimidine compounds. The MBC/MIC ratio indicated that the bactericidal effect was based on the record of Payveld [30].

Interestingly, the presence of a halogen particularly Cl or a methyl substituent in the *para* position on the imidazo[1,2-*a*]pyrimidine rings augments the activity threefold. Clearly, imidazo[1,2-*a*]pyrimidines having Br or F in the *para* position have moderate activity compared with compounds having a Cl substituent in the same position. It is worth noting that all imidazo[1,2-*a*]pyrimidines with halogenate substituents are more active against Gram-positive bacteria and *C. albicans* species. Further investigations are currently focused on anti-candidosis activity in our laboratory.

### 2.3. Molecular Docking Simulation

#### 2.3.1. Protein-Ligand Interaction

The docking analysis of the three compounds **3g**, **3k**, and **3j** with six X-ray crystal structures of the studied targets was performed, and the detailed results of the docking simulation (semi-flexible) are outlined in Table 5.

In order to estimate all possible interactions, the docking outputs generated by MOE software (https://www.chemcomp.com/en/Products.htm, accessed on 18 October 2024) were converted into (.pdb) files and visualized with the default parameters of the BIOVIA DS visualizer package (Dassault Systèmes BIOVIA, Discovery Studio Modeling Environment, 2020).

#### 2.3.2. Orientation and Bonding Interaction of the Compounds at the Active Site of Receptors

As shown in Table 5, we found that compounds **3g** and **3k** were predicted to be the strongest binders to the *S. aureus* target (PDB ID: 4URM) binder that forms the complexes, with the stability confirmed by the negative score energy of −5.241, and −4.871 Kcal/mol, respectively. Notably, the score value of compound **3g** is very close to that of the native ligand (XAM): −5.813 kcal/mol (Table 5).

**Figure 2:**
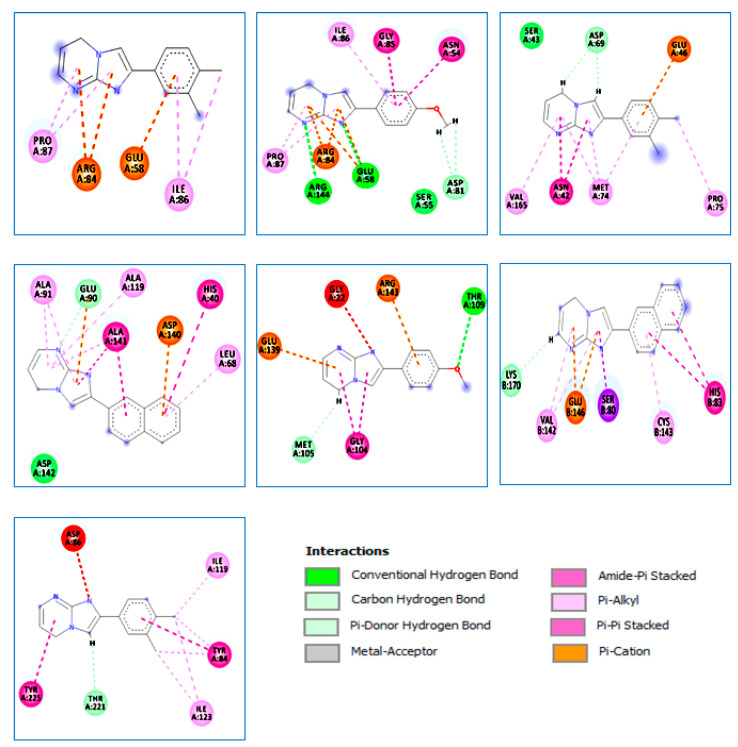
The 2D diagrams of the interaction between **3g** and *S. aureus* (PDB ID:4URM); **3k** and *S. aureus* (PDB ID:4URM); **3k** and *E. coli* (PDB ID:3FV5); **3j** and *B. cereus* (PDB ID:3DUW); **3g** and *B. subtilis* (PDB ID:2RHL); **3j** and *M. luteus* (PDB ID:3AQC); and **3k** and *C. albicans* (PDB ID:3Q70).

From the obtained results, it is apparent that compound **3g** formed four strong hydrogen bonds [29] with the active site residue of *S. aureus* target (PDB ID: 4URM); two conventional H-bonds, namely N/ARG144(A)-HH21/bond distance = 2.70Å and N/GLU58(A)-OE2/bond distance = 3.23Å; and two carbon–H-bonds, H/ASP81(A)-OD2/bond distance = 2.93Å and H/ASP81(A)-OD2/bond distance = 2.61Å. Four electrostatic interactions were observed with ARG84(A) and GLU58(A), while this compound formed seven hydrophobic interactions with the active site of the enzyme (Table 5; Figure 2).

**Figure 3:**
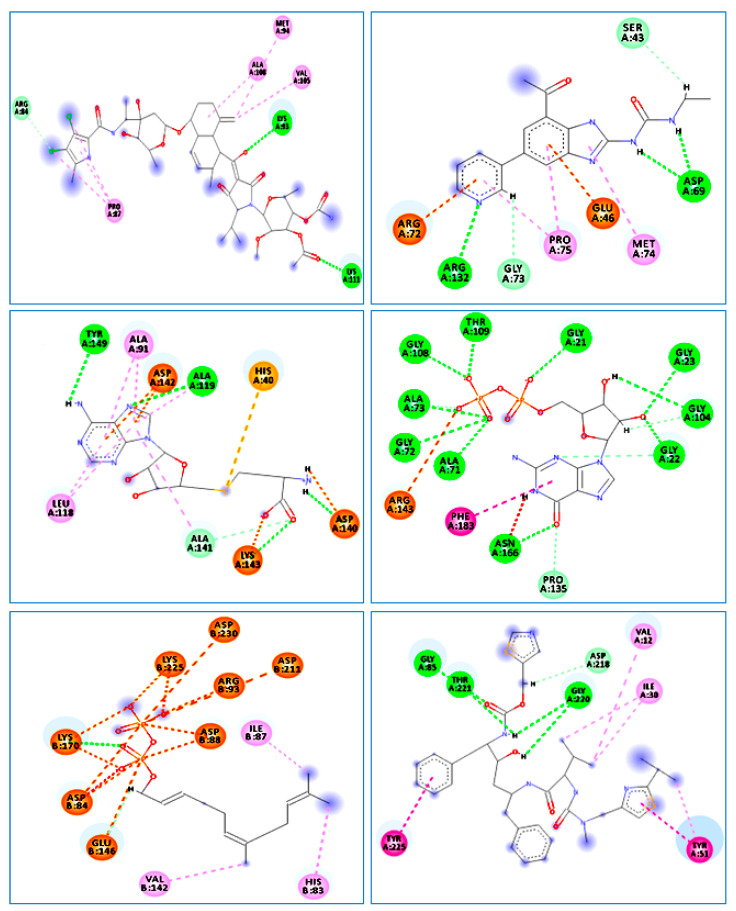
The 2D diagrams of the interaction between XAM and *S. aureus* (PDB ID:4URM); 1EU and *E. coli* (PDB ID:3FV5); SAH and *B. cereus* (PDB ID:3DUW); GDP and *B. subtilis* (PDB ID:2RHL); 2DE and *M. luteus* (PDB ID:3AQC); and RIT and *C. albicans* (PDB ID:3Q70)**.**

**Figure 4:**
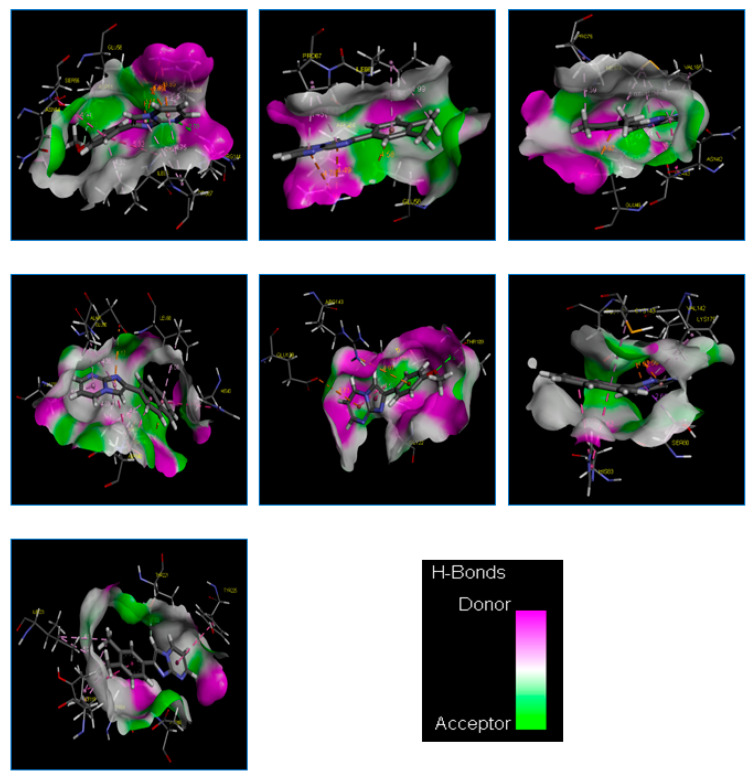
The 3D diagrams of the interaction between **3g** and *S. aureus* (PDB ID:4URM); **3k** and *S. aureus* (PDB ID:4URM); **3k** and *E. coli* (PDB ID:3FV5); **3j** and *B. cereus* (PDB ID:3DUW); **3g** and *B. subtilis* (PDB ID:2RHL); **3j** and *M. luteus* (PDB ID:3AQC); and **3k** and *C. albicans* (PDB ID: 3Q70).

Similarly, compound **3k** revealed three electrostatic interactions with the active site residue of the *S. aureus* target (PDB ID: 4URM) and four hydrophobic interactions with the pocket of the target (Table 5; Figure 2). In this regard, several studies [30,31,32] revealed that ARG84(A) plays an important role in the inhibition of the *S. aureus* (PDB ID: 4URM) target (Figure 3 and Figure 4).

Notably, the complex formed by compound **3g** exhibited the best negative energy value of −3.662 kcal/mol compared to the native ligand (1EU) (−3.552 kcal/mol) (Table 5). The docked conformation of compound **3g** with the *E. coli* target (PDB ID: 3FV5) is shown in Figure 2. We note that this compound formed two strong carbon–H-bonds: H/ ASP69(A)-OD2/bond distance = 2.13Å and H/ASP69(A)-OD2/bond distance = 2.41Å (Table 5; Figure 2). At the same time, compound **3g** formed one electrostatic interaction and seven hydrophobic interactions with the target active site residue of the target. Notably, several studies [33,34] have reported that ASN42(A), GLU46(A), and PRO75(A) play a central role in *E. coli* receptor (PDB ID: 3FV5) inhibition.

Compound **3j** formed the most stable complex with the *B. cereus* (PDB ID: 3DUW) target compared with the native ligand (SAH), as confirmed by the negative score energy values of −5.855 kcal/mol and −5.616 kcal/mol, respectively. This compound exhibited one strong carbon–hydrogen bond with the residue GLU90(A) (bond distance = 2.76Å Å) in addition to two electrostatic interactions with GLU90(A) and ASP140(A). Moreover, it exhibited ten hydrophobic interactions with the active site residue of the *B. cereus* target (PDB ID: 3DUW) (Table 5; Figure 2). Sokolova N. et al. [35] confirmed that these residues are critical in the formation of different interactions in the active site of this target.

The complex formed by compound **3g** exhibited the lowest binding energy score (−5.205 kcal/mol), which is very close to native ligand GDP 5 (−6.077 Kcal/mol) (Table 5). Furthermore, compound **3g** exhibited one strong hydrogen bond (conventional and carbon types) with the residues THR109(A) in MET105(A), respectively (bond distance = 2.95 Å and 2.70 Å). Two electrostatic interactions (Pi–cation and Pi–anion) were detected with GLU90(A) and ASP140(A), respectively. Consequently, two hydrophobic interactions were formed between this compound and the same residue, i.e., GLY104(A) (Table 5; Figure 2). These results have been confirmed by recent studies [34,35,36,37,38,39].

As presented in Table 5, we found that compound **3j** has a very close affinity with the *M. Luteus* (PDB ID: 3AQC) target compared to native ligand 2DE, with a value of −7.534 kcal/mol. This is through the formation of a stable complex with a negative energy score of −4.958 kcal/mol and a low RMSD value.

The docked conformation of compound **3** in the active site pocket of *M. luteus* (PDB ID: 3AQC) exhibited one strong H-bond formed between compound **3j** and the residue LYS170(B) (bond distance = 2.61Å). Also, two electrostatic interactions (Pi–anion) were observed, which were formed between this compound and the same residue GLU146(B). Additionally, compound **3j** exhibited six hydrophobic interactions with the different residues in the active site of the *M. luteus* (PDB ID: 3AQC) target (Table 5; Figure 2). It should be noted that these results were supported by previous studies [40,41,42].

Finally, the docking results of compound **3k** with the *C. albicans* (PDB ID: 3Q70) active pocket show that the complex binding score (S-score) was −5.040 kcal/mol, which is very close to the native ligand (3Q70-RIT), at −5.107 kcal/mol (Table 5). In addition, it should be noted that compound **3k** revealed one strong H-bond with the pocket of the *C. albicans* (PDB ID: 3Q70) receptor, formed between the compound and the residue THR221(A) (bond distance = 2.65Å). Moreover, seven hydrophobic interactions were detected between this compound and the different residues in the active site of the studied target (Table 5; Figure 2). These results were supported by Barakat et al. [43].

### 2.4. Drug-Likeness Evaluation

Different parameters of physicochemical properties were calculated in order to verify the drug-likeness rules by using Swiss ADME [44] online servers. All the results are listed in Table 6.

As shown in Table 6, it is apparent that in all compounds, namely **3g**, **3k**, and **3j**, the number of hydrogen bond donors was < 5 (n-HD: (0~7)), and the number of hydrogen bond acceptors was < 10 (n-HA: (0~10)). In addition, the molecular weight values of these compounds ranged from 100 to 500 g/mol, and the MLogP and WLogP values were <5. Also, nROTB values were <11, which denotes the flexibility of these compounds. On the other hand, all TPSA values obtained were less than 140 Å. According to these results, it can be concluded that all compounds satisfy all the criteria of drug-likeness without any violation of Lipinski, Veber, and Egan rules. Clearly, not all compounds pose problems with oral bioavailability and pharmacokinetic parameters.

### 2.5. ADME-T Properties

The absorption, distribution, metabolism, excretion, and toxicity parameters were calculated by using the online server pkCSM [45]. All the results are presented in Table 7.

From the analysis in Table 7, we can draw the following conclusions:

1. All compounds had Caco-2 values greater than −5.15 (>−5.15 cm/s). This means that these compounds exhibit good permeability. In addition, it is evident that all compounds had HIA values greater than 30%, indicating that the orally administered drug candidates are absorbed from the gastrointestinal system into the bloodstream of the human body.
2. All three compounds could penetrate the CNS, as confirmed by logPS values, which were in the range of −3 < logPS < −2. Additionally, the logBB values of compounds **3g**, **3k,** and **3j** were 0.311, 0.030, and 0.123, respectively. This indicates that compound **3g** readily crosses the blood–brain barrier and that compounds **3k** and **3j** are poorly distributed in the brain (Table 7).
3. A P450 CYP inhibitor test was performed to determine whether the drug blocked or decreased the activity of one or more isoforms of the CYP450 enzyme. Data from this table indicate that compounds **3g**, **3k**, and **3j** are inhibitors of the CYP1A2 isoform but not CYP2C19 inhibitors. In addition, these compounds are not CYP2D6 and CYP3A4 substrates.
4. Further analysis of the table showed that none of the compounds were likely to be an OCT2 substrate. Moreover, it can be clearly seen that these compounds have a low excretion clearance (<5 mL/min/kg) (Table 7).
5. Additionally, the selected compounds were neither hERG I nor hERG II inhibitors. However, none of these compounds posed a hepatotoxicity risk.

## 3. Experimental Section

### 3.1. Chemistry

#### 3.1.1. General Information

All solvents and reagents were purchased from Aldrich (St. Louis, MO, USA)/Merck (Rahway, NJ, USA) and used as received without any further purification. Routine monitoring of reactions was performed by thin-layer chromatography (TLC). Melting points were taken using a Bank Kofer Hrizbank apparatus standard WME 50–266 °C and were uncorrected. The ^1^H and ^13^C NMR spectra were recorded on Magritek SPINSOLVE 91 spectrometers (Magritek (Aachen, Germany)) at 91 and 23 MHz, respectively. Samples were recorded in DMSO-*d*_6_ solutions using TMS as an internal standard. The chemical shifts are expressed in δ units (ppm) and quoted downfield from TMS. Signals are abbreviated as follows: s, singlet; d, doublet; dd, doublet of doublet; m, multiplet. The electron impact mass spectra were obtained using a Bruker 320-MS instrument (Bruker, Billerica, MA, USA). Microwave irradiation was performed using a household microwave oven.

#### 3.1.2. General Procedure for the Synthesis of 2-arylimidazo[1,2-a]Pyrimidines (**3a**–**k**)

As described in Scheme 1, imidazo[1,2-*a*]pyrimidine derivatives (**3a**–**k**) were synthesized by a condensation reaction of 2-aminopyrimidine 1 (10 mmol) and several 2-bromoarylketones **2** (**a**–**k**) (10 mmol) without solvent, catalyzed by Al_2_O_3_, and irradiated using a domestic microwave for 90–300 s. The amount of the catalyst was 30% (*w*/*w*). The TLC method was used to track the reaction progress. Then, the crude was cooled at room temperature, filtered, and washed several times with a mixture of ethyl ether and ethanol to afford a pure solid with a good yield without the need for any purification.

##### 2-phenylimidazo[1,2-a]pyrimidines (**3a**)

Yield: 70%; light brown solid; mp 195–197 °C; ^1^H NMR (91 MHz, DMSO d_6_): 9.30 (dd, J = 6.7, 1.9 Hz, 1H), 8.98 −7.44 (m, 8H); ^13^C NMR (23 MHz, DMSO-*d*_6_): 157.29, 138.22, 131.07, 130.02, 126.81, 114.31; MS (ESI) calcd for C_12_H_9_N_3_ [M+H]^+^ 195.23; found 196. The characterization data are consistent with the results reported in the literature [28].

##### 2-(3-bromophenyl)imidazo[1,2-a]pyrimidine (**3b**)

Yield: 56%; light brown solid; mp 191–193 °C; ^1^H NMR (91 MHz, DMSO d_6_): 9.02 (dd, J = 6.7, 1.9 Hz, 1H), 8.59 (d, J = 9.6 Hz, 2H), 8.31 –7.17 (m, 7H); ^13^C NMR (23 MHz, DMSO-*d*_6_): 152.43, 142.05, 136.07, 134.74, 131.57, 131.40, 128.58, 125.02, 122.62, 110.37, 109.10; MS (ESI) calcd for C_12_H_8_BrN_3_ [M+H]^+^ 274.12; found 276. The obtained characterization results match those reported in the literature [18].

##### 2-(4-bromophenyl)imidazo[1,2-*a*]pyrimidine (**3c**)

Yield: 63%; white solid; mp 209–211 °C; ^1^H NMR (91 MHz, DMSO-*d*_6_): 9.21 (dd, J = 6.6, 1.9 Hz, 1H), 8.86–7.45 (m, 7H); MS (ESI) calcd for C_12_H_8_BrN_3_ [M+H]^+^ 274.12; found 276. The characterization results for this compound align with those available in the literature [9].

##### 2-(p-tolyl)imidazo[1,2-*a*]pyrimidine (**3d**)

Yield: 68%; pale solid; mp 229–231 °C; ^1^H NMR (91 MHz, DMSO *d*_6_): δ 9.48 (dd, J = 6.9, 1.7 Hz, 1H), 9.13–7.37 (m, 7H), 2.74–2.55 (m, 3H); ^13^C NMR (23 MHz, DMSO-d_6_): 156.90, 144.71, 140.87, 137.96, 136.74, 130.35, 126.51, 124.12, 114.14, 109.40, 21.16; MS (ESI) calcd for C_13_H_11_N_3_ [M+H]^+^ 209.25; found 210. The characterization data are consistent with the results reported in the literature [28].

##### 2-(2-methoxyphenyl)imidazo[1,2-*a*]pyrimidine (**3e**)

Yield: 57%; yellow solid; mp 264–266 °C; ^1^H NMR (91 MHz, DMSO-*d*_6_): 9.09–8.73 (m, 1H), 8.12–7.00 (m, 7H), 4.02 (s, 3H); ^13^C NMR (23 MHz, DMSO-*d*_6_): 157.00, 139.61, 133.97, 132.23, 131.69, 129.37, 128.17, 121.40, 117.66, 114.77, 114.18, 112.72, 56.53; MS (ESI) calcd for C_13_H_11_N_3_O [M+H]^+^ 225.25; found 226.

##### 2-(3-methoxyphenyl)imidazo[1,2-*a*]pyrimidine (**3f**)

Yield: 53%; brown solid; mp 219–221 °C; ^1^H NMR (91 MHz, DMSO-*d_6_*): 9.31 (dd, J = 6.7, 1.8 Hz, 1H), 9.13–6.86 (m, 7H), 3.85 (d, J = 1.7 Hz, 3H); ^13^C NMR (23 MHz, DMSO-*d*_6_): 160.25, 157.27, 144.80, 138.09, 136.54, 131.11, 128.26, 118.91, 116.77, 114.26, 111.90, 110.38, 56.03; MS (ESI) calcd for C_13_H_11_N_3_O [M+H]^+^ 225.25; found 226. The characterization results for this compound align with those available in the literature [9].

##### 2-(4-methoxyphenyl)imidazo[1,2-*a*]pyrimidine (**3g**)

Yield: 52%; yellow solid; mp 189–191 °C; ^1^H NMR (91 MHz, DMSO-*d*_6_): 9.52 (s, 1H), 8.15 –7.04 (m, 7H), 3.87 (d, J = 2.2 Hz, 3H); ^13^C NMR (23 MHz, DMSO-*d*_6_): 169.47, 164.43, 131.29, 127.30, 114.66, 107.81, 56.03; MS (ESI) calcd for C_13_H_11_N_3_O [M+H]^+^ 225.25; found 226. The characterization data are consistent with the results reported in the literature [28].

##### 2-(4-fluorophenyl)imidazo[1,2-*a*]pyrimidine (**3h**)

Yield: 63%; light brown solid; mp 230–232 °C; ^1^H NMR (91 MHz, DMSO-*d*_6_): 9.29 (dd, J = 6.7, 1.9 Hz, 1H), 8.96–7.26 (m, 7H); ^13^C NMR (23 MHz, DMSO-*d*_6_): 157.01, 144.81, 137.99, 135.90, 129.26, 128.89, 123.79, 117.39, 116.44, 114.06, 109.88; MS (ESI) calcd for C_12_H_8_FN_3_ [M+H]^+^ 213.22; found 214. The characterization data are consistent with the results reported in the literature [28].

##### 2-(4-chlorophenyl)imidazo[1,2-*a*]pyrimidine (**3i**)

Yield: 65%; light brown solid; mp 273–175 °C; ^1^H NMR (91 MHz, DMSO-*d*_6_): 9.25 (d, J = 6.9 Hz, 1H), 8.92–7.26 (m, 7H); ^13^C NMR (23 MHz, DMSO-*d*_6_): 156.39, 144.72, 137.56, 136.05, 128.91, 128.53, 123.83, 117.05, 116.09, 113.47, 109.32; MS (ESI) calcd for C_12_H_8_ClN_3_ [M+H]^+^ 229.67; found 214. The experimental data correspond well with those previously reported in the literature [10].

##### 2-(naphthalen-2-yl)imidazo[1,2-*a*]pyrimidine (**3j**)

Yield: 67%; pale solid; mp 249–251 °C; ^1^H NMR (91 MHz, DMSO-*d*_6_): 9.25 (dd, J = 6.8, 1.9 Hz, 1H), 8.96–7.46 (m, 10H); ^13^C NMR (23 MHz, DMSO-*d_6_*): 156.03, 145.74, 138.58, 137.64, 133.77, 133.21, 129.49, 128.84, 128.29, 127.85, 127.63, 126.06, 125.87, 123.79, 113.27, 110.08; MS (ESI) calcd for C_16_H_11_N_3_ [M+H]^+^ 245.29; found 246. The experimental data correspond well with those previously reported in the literature [10].

##### 2-(3,4-dimethylphenyl)imidazo[1,2-*a*]pyrimidine (**3k**)

Yield: 64%; light brown solid; mp 231–233 °C; ^1^H NMR (91 MHz, DMSO-*d*_6_): 9.27 (dd, J = 6.8, 1.9 Hz, 1H), 8.95–7.35 (m, 6H), 2.64–2.20 (m, 6H); ^13^C NMR (23 MHz, DMSO-d_6_): 156.47, 144.27, 139.32, 137.52, 136.42, 130.39, 127.05, 123.98, 123.63, 113.69, 108.88, 19.41, 19.32; MS (ESI) calcd for C_14_H_13_N_3_ [M+H]^+^ 223.28; found 224.

It should be noted that, even with the use of NMR spectrometers operating at 91 MHz and 23 MHz for ^1^H and ^13^C NMR, respectively, the products obtained correspond to those documented in the literature. The MS analysis revealed dominant peaks that precisely match the expected molar masses of the synthesized compounds, confirming their identification. Moreover, the ^1^H NMR spectra we acquired are identical to those stated in previous research, further confirming the chemical structure of the compounds. Although the ^13^C NMR spectra showed fewer peaks for some products—likely due to the performance and lower sensitivity of the instruments used—most of the signals align well with the literature data. The noted chemical shifts may indicate overlapping carbons, clarifying the absence of peaks that correspond to theoretical carbon positions. This supports compelling evidence for the effective synthesis of the desired compounds.

### 3.2. Biology

The antimicrobial potency of all imidazo[1,-2*a*]pyrimidine compounds was screened in vitro against 13 microorganisms, comprising 6 Gram-positive bacteria, 4 Gram-negative bacteria, and 3 yeasts (Table 8), using the disk diffusion method as described by Nariya et al. [46].

Briefly, 50 mg/mL of the synthesized compounds **3a**–**k** was dissolved into dimethylsulphoxide. Then, ten microliters of each solution was added to a disk. The inhibitory zones were calculated (mm) after 24 h of incubation at 37 °C. All the samples were taken in triplicates. The means and the SD were calculated using PAST 4.3 software.

The active compounds were determined by their minimum inhibitory concentration (MIC) on an ELISA plate using the method described by Premsai Rai et al. [47], with some modifications. Briefly, bacteria and yeast were inoculated into brain–heart infusion broth (BHIB) and Sabouraud broth, respectively, at 37 °C for 24 h. The optical density was adjusted to 5 × 106 colony-forming units/mL for bacteria and to 5 × 105 colony-forming units/mL for yeast, measured at 620 nm.

The graded concentrations of the active imidazo[1,2-*a*]pyrimidine compounds (0.039–20 mg/mL) and suspension microorganisms were added into the first ten wells of the ELISA plate to obtain 200 µL as the final concentration, and the samples were then incubated for 24 h at 37 °C. The MIC is expressed as the lowest concentration of compounds that showed no visible growth. The 11th well was considered a positive control, which contained the medium and inoculum. The 12th well was considered a negative control, which contained just the medium. The minimum bactericidal concentrations (MBCs) were assessed from the subcultured MIC wells. Ten microliters of each solution was added to Müller–Hinton agar or Sabouraud agar and incubated aerobically for 24 h at 37 °C. The lowest concentration with no growth, shown on the MHA plate, was recorded as the MBC.

### 3.3. In Vitro Assay for PDE5 Inhibitors

#### 3.3.1. Protein Expression and Purification

##### Ligands and Target Preparations

The 3D structures of the most active compounds (**3g**, **3k,** and, **3j**) were optimized using the semi-empirical method AM1 [48]**,** which was implemented in Hyperchem 8.0.8 software (Version 8.0.8, Hypercube, Gainesville, FL, USA). In addition, all these structures were converted into the .*mdb format in order to be used as input MOE docking.

Six crystal structures of targets were downloaded from Protein Data Bank (http://www.rcsb.org) and selected as antibacterial targets, namely gyrase B (PDB ID: 4URM) [49] from *Staphylococcus aureus*; the DNA topoisomerase complex (PDB ID: 3FV5) [48] of *E. coli*; BcOMT2 (PDB ID: 3DUW) [50] from *Bacillus cereus*; BsFtsZ (PDB ID: 2RHL) [51] from *Bacillus subtili*; and *hexaprenyl* diphosphate synthase (PDB ID: 3AQC) [39] from *Micrococcus luteus*. Secreted aspartic protease (PDB ID: 3Q70) [50] from *Candida albicans* was chosen as an antifungal target in order to understand the antibacterial activities of these compounds. Some information related to the target structures is provided in Table 9.

- Protocol and validation of the Docking method

Molecular docking simulations were performed using the MOE software [51] to discover and identify the binding interactions of compounds within the active site residues of six targets. The molecular docking protocol steps were followed and detailed in our previous research [52,53] using the following default parameters: Placement: Triangle Matcher, Rescoring 1: London dG. The scoring function used was London dG.

Furthermore, the method was validated by re-docking all the native ligands to their targets. The RSMD values of the obtained complexes (target-crystallized ligands) were less than 2.0 Å [54], implying that the docking method is accurate and successful.

- ADME-Tox evaluation

In order to validate the drug-likeness rules, namely Lipinski, Veber ,and Ghose, different parameters of physicochemical properties (TPSA, nROT, MW, LogP, number of hydrogen bond acceptors (nHAs), and number of hydrogen bond donors (nHDs)) were calculated using the SwissADME server (http://www.swissadme.ch/) [44].

On the other hand, the pkCSM server (http://biosig.unimelb.edu.au/pkcsm/prediction, accessed on 18 October 2024) [45] was used for the analysis of ADMET profiles by calculating the following parameters: absorption (Caco-2: colon adenocarcinoma and HIA: human intestinal absorption), distribution (CNS: central nervous system permeability and BBB: blood–brain barrier permeability), metabolism (CYP1A2 inhibitor, CYP2C19 inhibitor, and CYP2D6 inhibitor), excretion (Renal OCT2 substrate: organic cation transporter 2 and total clearance), and toxicity (hERG: human ether-à-go-go-related gene and hepatotoxicity).

## 4. Conclusions

The reported method for the synthesis of imidazo[1,2-*a*]pyrimidine compounds assisted by microwave irradiation offers several advantages with respect to the mechano-synthesis and thermal treatment methods, such as higher yields, very short reaction times, and easy work-up procedure. In addition, the condensation reaction to produce imidazo[1,2-*a*]pyrimidine derivatives assisted by microwave irradiation and catalyzed by Al_2_O_3_ without any solvent was performed under more environmentally friendly conditions compared to other evaluation methods. In this study, all the compounds were tested, and they showed good activity against Gram-positive bacteria and yeasts but not against Gram-negative bacteria. Some of the compounds exhibited better antimicrobial activity compared to others.

Furthermore, the molecular docking study showed that compounds **3g**, **3k,** and **3j** have high affinity for the binding site of the antimicrobial target, which was confirmed by the lowest energy score with good binding modes, leading to the formation of different interaction types. Additionally, in silico ADME and toxicity predictions were performed on these compounds, and most of them were found to comply with the Lipinski, Veber, and Egan rules with good drug-likeness, some oral bioavailability properties, and good pharmacokinetic profiles. This study confirms that these derivatives are not hepatotoxic and are not carcinogenic.

Finally, this research identifies new imidazo[1,2-*a*]pyrimidines derivatives that may aid in the design and development of novel antibacterial agents.

## Figures and Tables

**Table 1:** Influence of catalyst load on the imidazo[1,2-*a*]pyrimidine synthesis.

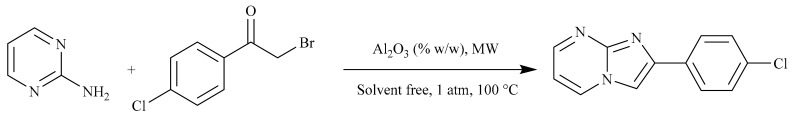			
Entry	Catalyst (% *w*/*w*)	Yield (%)	Reaction Time (s)
**1**	10	26	150
**2**	20	45	120
**3**	30	65	90
**4**	40	63	90
**5**	50	34	120

**Table 2:** Structure and physical parameters of the synthesized imidazo[1,2-*a*]pyrimidine derivatives.

Entry	Structure	Yield (%)	Yield (Ref %)	Melting Point (°C)	Melting Point (Ref °C)	Reaction Time (s)
**3a**	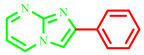	70	80 [28]	195–197	190–192 [28]	240
**3b**	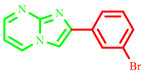	56	80 [18]	191–193	220 [18]	300
**3c**	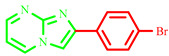	63	94 [9]	209–211	212–214 [9]	90
**3d**	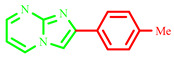	68	75 [28]	229–231	236–238 [28]	150
**3e**	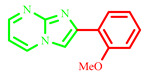	57	-----	264–266	Not described	200
**3f**	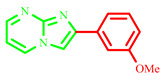	53	90 [9]	219–221	222–224 [9]	200
**3g**	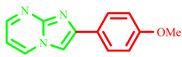	52	70 [28]	189–191	188–190 [28]	200
**3h**	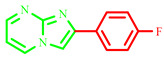	63	85 [28]	230–232	236–238 [28]	110
**3i**	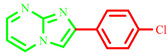	65	83 [10]	273–275	267–269 [10]	90
**3j**	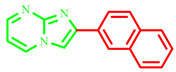	67	91 [10]	249–251	276–278 [10]	220
**3k**	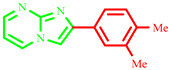	64	-----	231–233	Not described	180

**Table 3:** Zones of inhibition (in mm) of compounds **3a**–**k** (concentration used: 50 mg/mL of DMSO).

Compounds	Gram-Positive Bacteria						
	*S. aureus*	*M. luteus*	*L. monocytogenes*	*E. faecalis*	*B. cereus*	*B. subtilis*	
**3a**	8.6 ± 0.3	8.3 ± 0.3	/	/	/	8.3 ± 0.3	
**3b**	8.3 ± 0.3	/	/	/	/	/	
**3c**	12.3 ± 0.3	15.6 ± 0.3	/	/	11 ± 0.6	9.3 ± 0.3	
**3d**	/	/		/	/	/	
**3e**	8.6 ± 0,3	9.6 ± 0.3		7 ± 0	7 ± 0	/	
**3f**	8.3 ± 0.3	8 ± 0.0		/	10.6 ± 0.3	9.3 ± 0.3	
**3g**	12.6 ± 0.3	/	/	/	/	19.3 ± 0.3	
**3h**	7.3 ± 0.3	/		/	7 ±0.0	7 ± 0.0	
**3i**	8.6 ± 0.3	/	/	/	/	7.6 ± 0.3	
**3j**	12.3 ± 0.3	13.3 ± 0.3		/	11.3 ± 0.3	8.6 ± 0.3	
**3k**	10.6 ± 0.3	9.3 ± 0.3		/	12.3 ± 0.3	9.3 ± 0.3	
	**Gram-Negative Bacteria**				**Yeasts**		
**Compounds**	***E. coli***	***K. pneumoniae***	***P. areuginosa***	***S. typhymerium***	***C. albicans ATCC 26790***	***C. albicans ATCC 10231***	***C. albicans IPP 444***
**3a**	8.6 ± 0.3	/	/	/	9.6 ± 0.3	15 ± 1	/
**3b**	/	/	/	/	/	/	/
**3c**	6 ± 0.0	/	/	/	13 ± 0.57	18.3 ± 0.6	/
**3d**	/	/	/	/	8.6 ± 0.3	/	/
**3e**	/	/	/	/	/	/	/
**3f**	/	/	/	/	10 ± 0.5	11.6 ± 0.3	/
**3g**	/	/	/	/	13 ± 0.5	19.3 ± 0.3	/
**3h**	/	/	/	/	14 ± 0.57	/	/
**3i**	/	/	/	/	/	/	/
**3j**	/	/	/	/	15 ± 0.3	12.3 ± 0.5	14 ± 0.5
**3k**	/	/	/	/	15.6 ± 0.3	12.6 ± 0.3	14.6 ± 0.3

**Table 4:** In vitro MIC and MBC (mg/mL) of synthesized active compounds.

Product		*S. aureus*	*M. luteus*	*B. cereus*	*B. subtilis*	*C. albicans 26790*	*C. albicans 10231*	*C. albicans IPP 444*
**3a**	MIC	/	/	/	/	/	5	/
	MBC	/	/	/	/	/	10	/
**3c**	MIC	/	/	/	/	5	5	/
	MBC	/	/	/	/	10	10	/
**3g**	MIC	5	/	/	2.5	5	2.5	/
	MBC	10	/	/	2.5	10	5	/
**3h**	MIC	/	/	/	/	5	/	/
	MBC	/	/	/	/	5	/	/
**3j**	MIC	10	5	/	/	2.5	5	5
	MBC	10	10	/	/	5	5	10
**3k**	MIC	/	/	20	/	2.5	5	2.5
	MBC	/	/	20	/	2.5	10	5

**Table 5:** Docking score energy, RMSD values, and interactions between the most active compounds and native ligands with active site residues of antibacterial and antifungal targets.

*S. aureus * (PDB ID: 4URM)								
Compounds	S-Score (kcal/mol)	RMSD (Å)	Bonds Between Atoms of Compounds and Active Site Residues					
			Atom of Compound	Involved Receptor Atoms	Involved Receptor Residues	Category	Type of Interaction	Distance (Å)
**3g**	−5.241	2.016	N	HH21	ARG144(A)	Hydrogen Bond	Conventional H-Bond	2.70
			N	OE2	GLU58(A)	Hydrogen Bond	Conventional H-Bond	3.23
			H	OD2	ASP81(A)	Hydrogen Bond	Carbon–H-Bond	2.93
			H	OD2	ASP81(A)	Hydrogen Bond	Carbon–H-Bond	2.61
			/	NH2	ARG84(A)	Electrostatic	Pi–Cation	3.84
			/	NH2	ARG84(A)	Electrostatic	Pi–Cation	4.46
			/	OE1	GLU58(A)	Electrostatic	Pi–Anion	4.48
			/	OE2	GLU58(A)	Electrostatic	Pi–Anion	3.64
			/	C	ASN54(A)	Hydrophobic	Amide–Pi Stacked	4.94
			/	C	GLY85(A)	Hydrophobic	Amide–Pi Stacked	5.31
			/	/	ARG84(A)	Hydrophobic	Pi–Alkyl	5.11
			/	/	PRO87(A)	Hydrophobic	Pi–Alkyl	4.75
			/	/	ARG84(A)	Hydrophobic	Pi–Alkyl	5.42
			/	/	PRO87(A)	Hydrophobic	Pi–Alkyl	5.13
			/	/	ILE86(A)	Hydrophobic	Pi–Alkyl	4.31
**3k**	−4.871	1.898	/	NH2	ARG84(A)	Electrostatic	Pi–Cation	4.78
			/	NH2	ARG84(A)	Electrostatic	Pi–Cation	4.49
			/	OE1	GLU58(A)	Electrostatic	Pi-Anion	4.57
			C	/	ILE86(A)	Hydrophobic	Alkyl	4.99
			/	/	PRO87(A)	Hydrophobic	Pi–Alkyl	4.44
			/	/	PRO87(A)	Hydrophobic	Pi–Alkyl	4.42
			/	/	ILE86(A)	Hydrophobic	Pi–Alkyl	4.53
**Native** **ligand (XAM)**	−5.813	1.408	OBB	HZ3	LYS93(A)	Hydrogen Bond	Conventional H-Bond	2.19
			OBY	HZ2	LYS11(A)	Hydrogen Bond	Conventional H-Bond	1.99
			CLX	HD3	ARG84(A)	Hydrogen Bond	Carbon–H-Bond	3.02
			/	/	ALA108(A)	Hydrophobic	Alkyl	4.86
			CLW	/	PRO87(A)	Hydrophobic	Alkyl	5.09
			CLX	/	ARG84(A)	Hydrophobic	Alkyl	3.98
			CLX	/	PRO87(A)	Hydrophobic	Alkyl	4.70
			CAK	/	MET94(A)	Hydrophobic	Alkyl	5.09
			CAK	/	VAL105(A)	Hydrophobic	Alkyl	4.36
			/	/	PRO87(A)	Hydrophobic	Pi–Alkyl	4.22
***E. coli *** **(PDB ID: ** **3FV5** **)**								
**3k**	−3.662	0.884	H	OD2	ASP69(A)	Hydrogen Bond	Carbon–H-Bond	2.13
			H	OD2	ASP69(A)	Hydrogen Bond	Carbon–H-Bond	2.41
			/	OE2	GLU46(A)	Electrostatic	Pi–Anion	3.96
			/	C	ASN42(A)	Hydrophobic	Amide–Pi Stacked	4.27
			/	C	ASN42(A)	Hydrophobic	Amide–Pi Stacked	4.21
			C	/	PRO75(A)	Hydrophobic	Alkyl	4.58
			/	/	MET74(A)	Hydrophobic	Pi–Alkyl	4.78
			/	/	VAL165(A)	Hydrophobic	Pi–Alkyl	5.19
			/	/	MET74(A)	Hydrophobic	Pi–Alkyl	4.03
			/	/	MET74(A)	Hydrophobic	Pi–Alkyl	5.06
**Native** **Ligand** **(1EU)**	−3.552	1.362	N24	HH11	ARG132(A)	Hydrogen Bond	Conventional H-Bond	2.50
			H6	OD2	ASP69(A)	Hydrogen Bond	Conventional H-Bond	1.76
			H7	OD2	ASP69(A)	Hydrogen Bond	Conventional H-Bond	2.35
			H5	OG	SER43(A)	Hydrogen Bond	Carbon–H-Bond	2.89
			H17	O	GLY73(A)	Hydrogen Bond	Carbon–H-Bond	2.63
			/	NH1	ARG72(A)	Electrostatic	Pi–Cation	3.63
			/	OE2	GLU46(A)	Electrostatic	Pi–Anion	4.09
			/	/	MET74(A)	Hydrophobic	Pi–Alkyl	4.51
			/	/	PRO75(A)	Hydrophobic	Pi–Alkyl	4.91
			/	/	ARG72(A)	Hydrophobic	Pi–Alkyl	5.09
			/	/	PRO75(A)	Hydrophobic	Pi–Alkyl	4.70
***B. Cereus *** **(PDB ID: ** **3DUW** **)**								
**3j**	−5.855	1.164	N	HA	GLU90(A)	Hydrogen Bond	Carbon–H-Bond	2.76
			/	OE2	GLU90(A)	Electrostatic	Pi–Anion	4.16
			/	OD1	ASP140(A)	Electrostatic	Pi–Anion	4.36
			/	/	HIS40(A)	Hydrophobic	Pi–Pi Stacked	5.95
			/	C	ALA141(A)	Hydrophobic	Amide–Pi Stacked	4.79
			/	C	ALA141(A)	Hydrophobic	Amide–Pi Stacked	4.17
			/	/	ALA91(A)	Hydrophobic	Pi–Alkyl	3.52
			/	/	ALA119(A)	Hydrophobic	Pi–Alkyl	5.01
			/	/	ALA141(A)	Hydrophobic	Pi–Alkyl	4.08
			/	/	ALA91(A)	Hydrophobic	Pi–Alkyl	4.36
			/	/	ALA141(A)	Hydrophobic	Pi–Alkyl	3.69
			/	/	ALA141(A)	Hydrophobic	Pi–Alkyl	4.62
			/	/	LEU68(A)	Hydrophobic	Pi–Alkyl	5.08
**Native** **Ligand (SAH)**	−5.616	1.623	N7	H	ALA119(A)	Hydrogen Bond	Conventional H-Bond	2.56
			O	HZ1	LYS143(A)	Hydrogen Bond	Conventional H-Bond	2.19
			HN1	O	ASP140(A)	Hydrogen Bond	Conventional H-Bond	1.88
			HN61	OH	TYR149(A)	Hydrogen Bond	Conventional H-Bond	2.78
			O	HA	ALA141(A)	Hydrogen Bond	Carbon–H-Bond	2.93
			/	OD1	ASP142(A)	Electrostatic	Pi–Anion	3.36
			/	OD2	ASP142(A)	Electrostatic	Pi–Anion	3.14
			HN2	OD2	ASP140(A)	Electrostatic	Attractive charge	2.00
			OXT	NZ	LYS143(A)	Electrostatic	Attractive charge	4.31
			SD	/	HIS40(A)	Other	Pi–Sulfur	5.65
			/	/	ALA91(A)	Hydrophobic	Pi–Alkyl	3.80
			/	/	LEU118(A)	Hydrophobic	Pi–Alkyl	5.30
			/	/	ALA119(A)	Hydrophobic	Pi–Alkyl	5.09
			/	/	ALA141(A)	Hydrophobic	Pi–Alkyl	4.75
			/	/	ALA91(A)	Hydrophobic	Pi–Alkyl	4.69
			/	/	LEU118(A)	Hydrophobic	Pi–Alkyl	4.66
***B. Subtilis* (PDB ID: ** **2RHL)**								
**3g**	−5.205	1. 634	O	HG1	THR109(A)	Hydrogen Bond	Conventional H-Bond	2.95
			H	O	MET105(A)	Hydrogen Bond	Carbon–H-Bond	2.70
			/	NH1	ARG143(A)	Electrostatic	Pi–Cation	4.97
			/	OE1	GLU139(A)	Electrostatic	Pi–Anion	4.72
			/	C	GLY104(A)	Hydrophobic	Amide–Pi Stacked	4.68
			/	C	GLY104(A)	Hydrophobic	Amide–Pi Stacked	4.52
**Native** **Ligand (GDP)**	−6.077	1.336	O1A	H	GLY21(A)	Hydrogen Bond	Conventional H-Bond	2.01
			O2	H	GLY22(A)	Hydrogen Bond	Conventional H-Bond	1.82
			O2	H	GLY23(A)	Hydrogen Bond	Conventional H-Bond	2.76
			O3B	H	ALA71(A)	Hydrogen Bond	Conventional H-Bond	2.19
			O3B	H	ALA72(A)	Hydrogen Bond	Conventional H-Bond	2.65
			O3B	H	ALA73(A)	Hydrogen Bond	Conventional H-Bond	2.13
			O2B	H	GLY108(A)	Hydrogen Bond	Conventional H-Bond	2.30
			O2B	H	THR109(A)	Hydrogen Bond	Conventional H-Bond	1.86
			O2B	HG1	THR109(A)	Hydrogen Bond	Conventional H-Bond	2.30
			O6	HD21	ASN166(A)	Hydrogen Bond	Conventional H-Bond	2.19
			HO3	O	GLY104(A)	Hydrogen Bond	Conventional H-Bond	2.90
			O2	HA2	GLY22(A)	Hydrogen Bond	Carbon–H-Bond	3.03
			N3	HA2	GLY22(A)	Hydrogen Bond	Carbon–H-Bond	2.57
			O1B	HA	ALA73(A)	Hydrogen Bond	Carbon–H-Bond	2.65
			O2B	HA2	GLY108(A)	Hydrogen Bond	Carbon–H-Bond	2.98
			O6	HA	PRO135(A)	Hydrogen Bond	Carbon–H-Bond	2.59
			H2	O	GLY104(A)	Hydrogen Bond	Carbon–H-Bond	2.16
			O1B	NH2	ARG143(A)	Electrostatic	Attractive charge	4.10
			/	/	PHE183(A)	Hydrophobic	Pi–Pi Shaped	4.82
***M. luteus* (PDB ID: ** **3AQC)**								
**3j**	−4.958	1.424	H	O	LYS170(B)	Hydrogen Bond	Carbon–H-Bond	2.61
			/	OE2	GLU146(B)	Electrostatic	Pi–Anion	3.69
			/	OE2	GLU146(B)	Electrostatic	Pi–Anion	4.02
			/	HB2	SER80(B)	Hydrophobic	Pi–Sigma	2.66
			/	/	HIS83(B)	Hydrophobic	Pi-Pi Stacked	5.61
			/	/	HIS83(B)	Hydrophobic	Pi-Pi Stacked	5.72
			/	/	VAL142(B)	Hydrophobic	Pi–Alkyl	5.49
			/	/	VAL142(B)	Hydrophobic	Pi–Alkyl	5.31
			/	/	CYS143(B)	Hydrophobic	Pi–Alkyl	5.11
**Native** **Ligand (2DE)**	−7.534	1.144	O1A	HZ2	LYS170(B)	Hydrogen Bond	Conventional H-Bond	1.71
			O1A	HE3	LYS170(B)	Hydrogen Bond	Carbon–H-Bond	2.87
			H2	OE2	GLU146(B)	Hydrogen Bond	Carbon–H-Bond	2.47
			O3B	HZ1	LYS225(B)	Electrostatic	Attractive charge	2.13
			O3B	NH1	ARG93(B)	Electrostatic	Attractive charge	4.10
			O2B	NZ	LYS170(B)	Electrostatic	Attractive charge	4.96
			O2B	NZ	LYS170(B)	Electrostatic	Attractive charge	4.58
			O2B	NZ	LYS225(B)	Electrostatic	Attractive charge	3.84
			PA	OD2	ASP84(B)	Electrostatic	Attractive charge	3.69
			PA	OD2	ASP88(B)	Electrostatic	Attractive charge	3.87
			PA	OE2	GLU146(B)	Electrostatic	Attractive charge	5.36
			PB	OD2	ASP84(B)	Electrostatic	Attractive charge	4.52
			PB	OD2	ASP88(B)	Electrostatic	Attractive charge	4.40
			PB	OD2	ASP211(B)	Electrostatic	Attractive charge	5.34
			PB	OD1	ASP230(B)	Electrostatic	Attractive charge	5.06
			C10	/	VAL142(B)	Hydrophobic	Alkyl	5.13
			C14	/	ILE87(B)	Hydrophobic	Alkyl	5.25
			C15	/	HIS83(B)	Hydrophobic	Pi–Alkyl	5.30
***C. albicans* (PDB ID: ** **3Q70)**								
**3k**	−5.040	1.501	H	OG1	THR221(A)	Hydrogen Bond	Carbon–H-Bond	2.65
			/	/	TYR84(A)	Hydrophobic	Pi–Pi Stacked	5.27
			/	/	TYR225(A)	Hydrophobic	Pi–Pi Stacked	5.02
			C	/	ILE119(A)	Hydrophobic	Alkyl	4.00
			C	/	ILE123(A)	Hydrophobic	Alkyl	5.22
			C	/	ILE123(A)	Hydrophobic	Alkyl	4.97
			C	/	TYR84(A)	Hydrophobic	Pi–Alkyl	4.43
			C	/	TYR84(A)	Hydrophobic	Pi–Alkyl	4.56
**Native** **ligand (RIT)**	−5.107	1.464	O24	HN	GLY85(A)	Hydrogen Bond	Conventional H-Bond	2.42
			H5	O	GLY220(A)	Hydrogen Bond	Conventional H-Bond	2.87
			H5	OG1	THR221(A)	Hydrogen Bond	Conventional H-Bond	2.54
			H18	O	GLY220(A)	Hydrogen Bond	Conventional H-Bond	2.38
			H4	OD2	ASP218(A)	Hydrogen Bond	Carbon H-Bond	3.09
			/	/	TYR225(A)	Hydrophobic	Pi–Pi Stacked	4.90
			/	/	TYR51(A)	Hydrophobic	Pi–Pi Stacked	4.40
			C64	/	ILE30(A)	Hydrophobic	Alkyl	5.20
			C68	/	VAL12(A)	Hydrophobic	Alkyl	5.49
			C68	/	ILE30(A)	Hydrophobic	Alkyl	5.29
			C86	/	TYR51(A)	Hydrophobic	Pi–Alkyl	4.03

**Table 6:** Physicochemical properties and drug-likeness predictions of compounds **3g**, **3k,** and **3j**.

Compounds	Physicochemical Properties						Medicinal Chemistry		
							Drug-Likeness Rules		
	TPSA (Å^2^)	n-ROT	MW (g/mol)	MLog P	n-HA	n-HD	Lipinski	Veber	Egan
				WLogP					
	(0~140)	(0~11)	(100~500)	(0~5)	(0~12)	(0~7)			
**3g**	39.42	2	225.25	1.49	3	0	Accepted	Accepted	Accepted
				2.40					
**3k**	30.19	1	223.27	2.33	2	0	Accepted	Accepted	Accepted
				3.01					
**3j**	30.19	1	245.28	2.65	2	0	Accepted	Accepted	Accepted
				3.55					

TPSA: topological polar surface area, n-ROT: number of rotatable bonds, MW: molecular weight, Log P: logarithm of partition coefficient of compound between n-octanol and water, n-HA: number of hydrogen bond acceptors, n-HD: Number of hydrogen bonds donors.

**Table 7:** ADMET/pharmacokinetic properties of compounds **3g**, **3k,** and **3j**.

ADME	Parameters	3g	3k	3j
Absorption	Caco2 (10^−6^ cm/s)	1.178	1.417	1.526
	HIA (%)	99.207	97.682	97.629
Distribution	CNS (log PS)	−1.800	−1.692	−1.462
	BBB (log BB)	0.311	0.030	0.123
Metabolism	CYP1A2 inhibitor	Yes	Yes	Yes
	CYP2C19 Inhibitor	No	No	No
	CYP2D6 substrate	No	No	No
	CYP3A4 substrate	No	No	No
Excretion	Renal OCT2 substrate	No	No	No
	Total Clearance (log mL/min/kg)	0.744	0.780	0.737
Toxicity	hERG I and II inhibitors	No	No	No
	Hepatotoxicity	No	No	No

Caco-2: colon adenocarcinoma, HIA: human intestinal absorption, CNS: central nervous system permeability, BBB: blood–brain barrier permeability, Renal OCT2 substrate: organic cation transporter 2, hERG: human ether-à-go-go-related gene.

**Table 8:** Microorganism strains and their ATCC numbers.

Gram-Positive Bacteria					
*Staphylococcus aureus* ATCC 25923	*Micrococcus* *luteus* ATCC 9341	*Listeria monocytogenes* ATCC 15313	*Bacillus* *cereus* ATCC 10876	*Bacillus subtilis* ATCC6633	*Enterococcus faecalis* ATCC29212
**Gram-Negative Bacteria**					
*Escherichia coli* ATCC 25912	*Pseudomonas aeruginosa* ATCC 27853	*Klebsiella pneumoniae* ATCC 700603		*Salmonella typhimurium* ATCC 13311	
**Yeasts**					
*Candida albicans* ATCC 26790		*Candida albicans* ATCC 10231		*Candida albicans* IPP 444	

**Table 9:** Data related to the six targets studied.

Targets PDB	Methods	Organism	Chain	Sequence Length	Resolution (Å)	Native Ligands
4URM	X-ray diffraction	*Staphylococcus aureus*	A, B	123	1.85	9JH
3FV5	X-ray diffraction	*E. coli K-12*	A, B	201	1.80	1EU
3DUW	X-ray diffraction	*B. cereus*	A, B	223	1.20	SAH
2RHL	X-ray diffraction	*Bacillus subtilis*	A, B	325	2.45	GDP
3AQC	X-ray diffraction	*M. luteus*	A, B, C, D	325	2.61	2DE
3Q70	X-ray diffraction	*C. albicans*	A	342	1.40	RIT

## Data Availability

Data is contained within the article or Supplementary Materials.

## Supplementary Materials

The following supporting information can be downloaded at: https://www.mdpi.com/article/10.3390/molecules29215058/s1, compounds **3a**–**3k**.
