# Supplementary material for: Design, Synthesis, Molecular Docking, and ADME-Tox Investigations of Imidazo[1,2-a]Pyrimidines Derivatives as Antimicrobial Agents
Source: Molecules. 2024 Oct 26;29(21):5058. doi: 10.3390/molecules29215058 (PMC11547262; doi:10.3390/molecules29215058)

Supplementary Materials

Design, Synthesis, Molecular Docking, and ADME-Tox Investigations of Imidazo[1,2-a]Pyrimidines Derivatives as Antimicrobial Agents

Djamila Benzenine [1,2], Ismail Daoud [3,4], Nadia Aissaoui [5], Zahira Kibou [1,2,*], Julio A. Seijas [6,*], M. Pilar Vázquez-Tato [6], Chewki Ziani-Cherif [1], Lahcen Belarbi [2,7] and Noureddine Choukchou-Braham [1]

3a

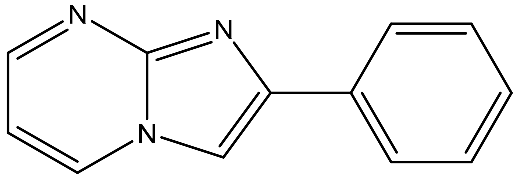

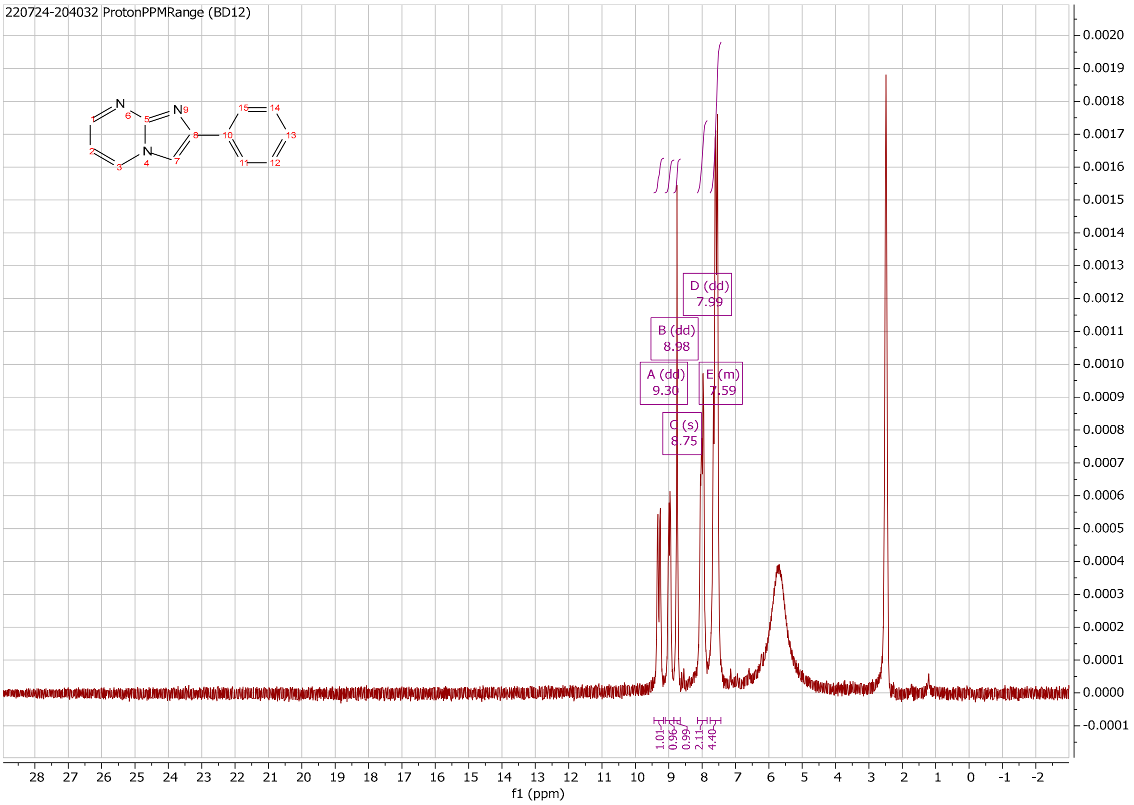
220724-204032 ProtonPPMRange (BD12)
A (dd) 9.30
B (dd) 8.98
C (s) 8.75
D (dd) 7.99
E (m) 7.59
1.01
0.96
0.99
2.11
4.40
f1 (ppm)

magritek

220724-205357 APT (BD12APT)

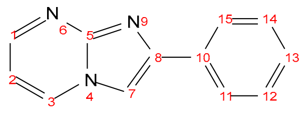

Parámetro	Valor
1 Nombre del Archivo de Datos	C:/ Data/ SpinsolveExpert/ 2022/ 07/ 24/ 220724-205357 APT (BD12APT)/ data.1d
2 Título	220724-205357 APT (BD12APT)
3 Comentario	
4 Origen	
5 Propietario	
6 Sitio	
7 Instrumento	
8 Autor	
9 Disolvente	
10 Temperatura	
11 Secuencia del Pulso	
12 Experimento	1D
13 Sonda	

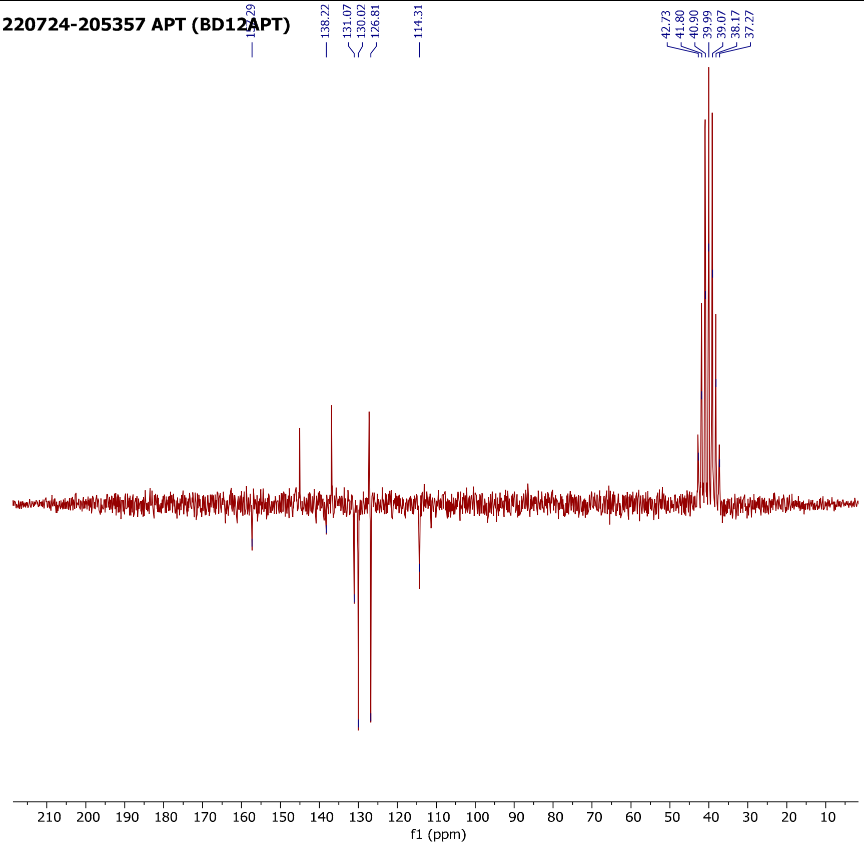

Analysis Info

Sample Name	3 APCI BD12 195_41_01_967.d	Acquisition Date	08/04/2022 10:53:10
Method	967.m	Instrument	amaZon ETD

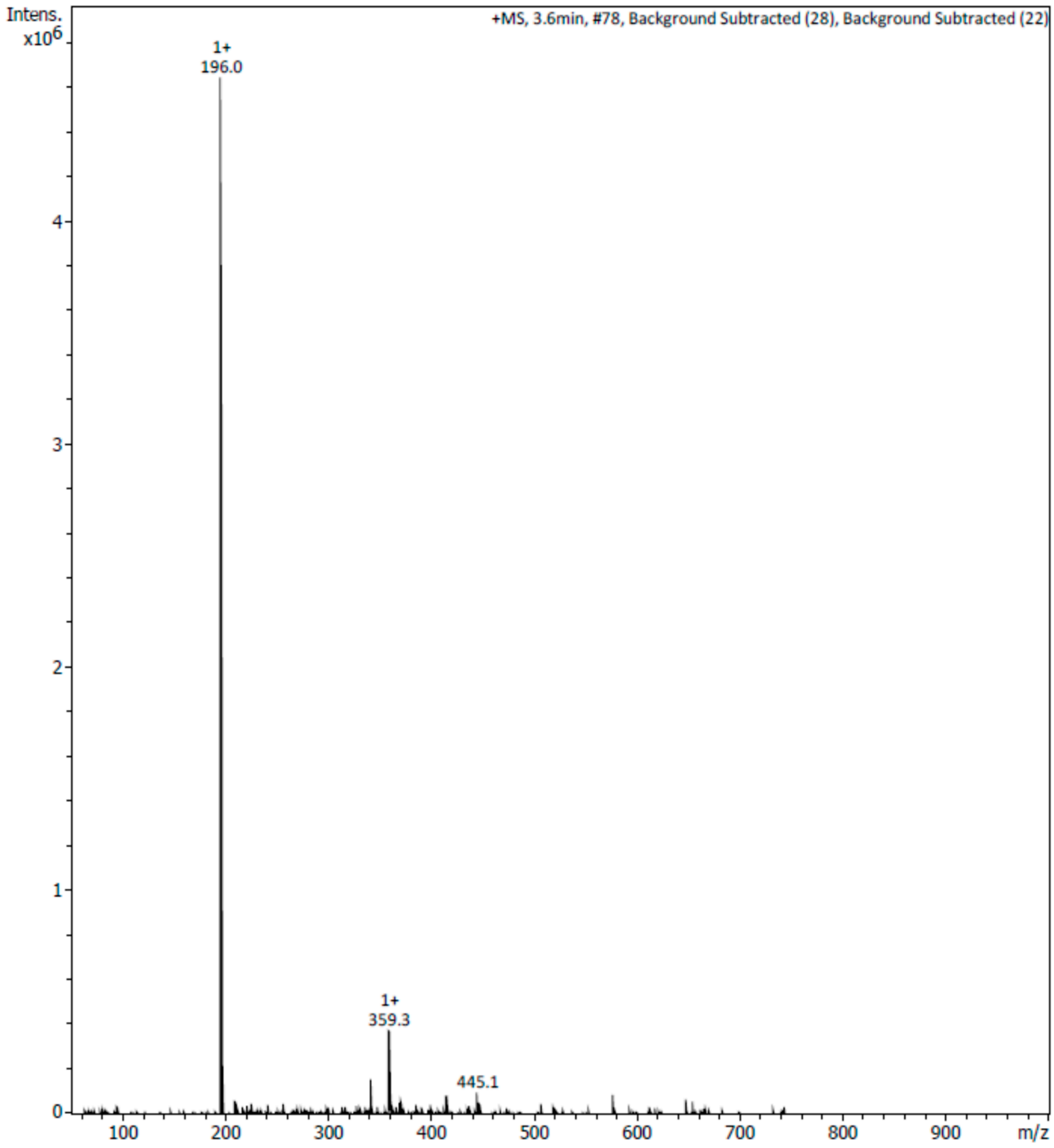

3b

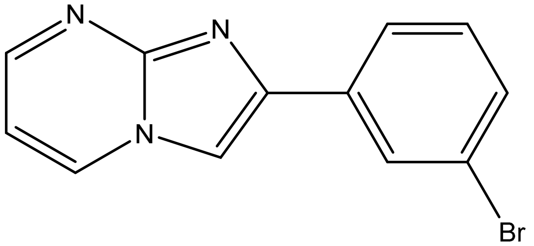

N
N
N
Br

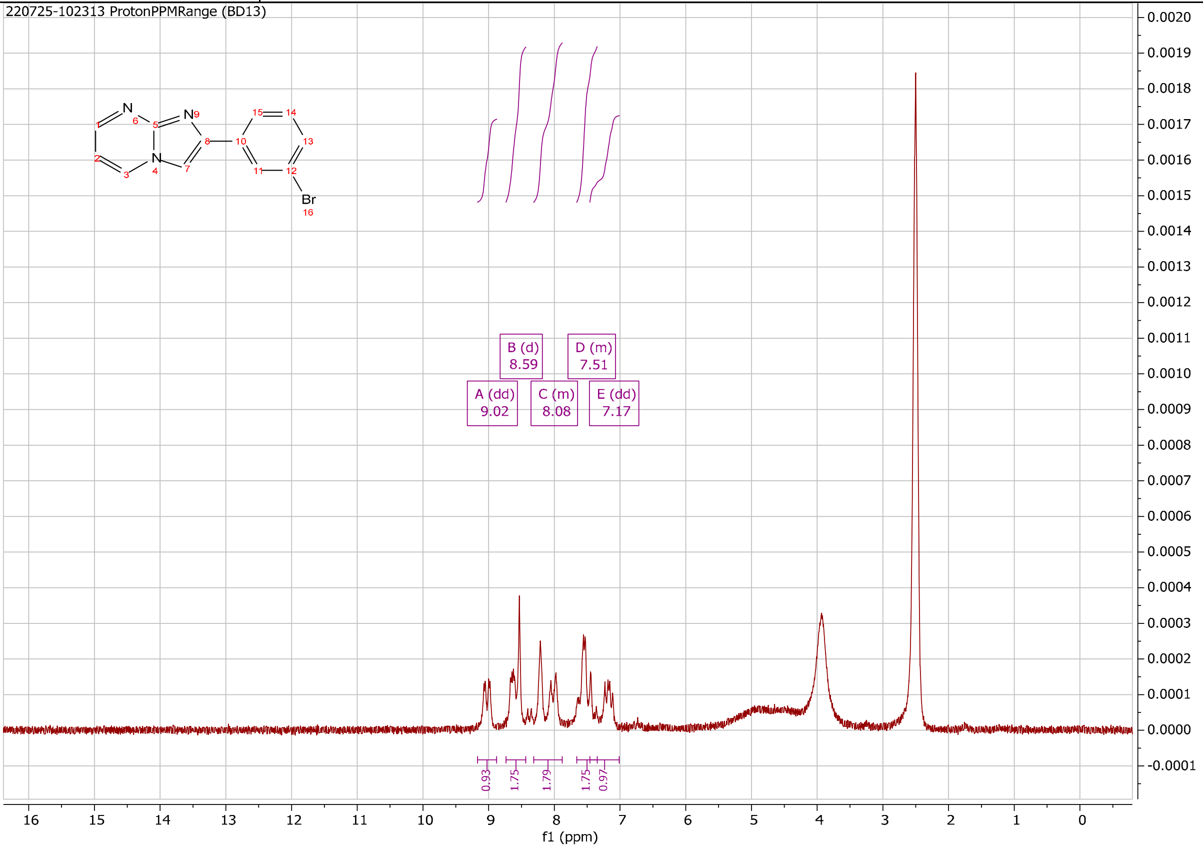

220725-102313 ProtonPPMRange (BD13)
A (dd)
9.02
B (d)
8.59
C (m)
8.08
D (m)
7.51
E (dd)
7.17
0.93
1.75
1.79
1.75
0.97
f1 (ppm)

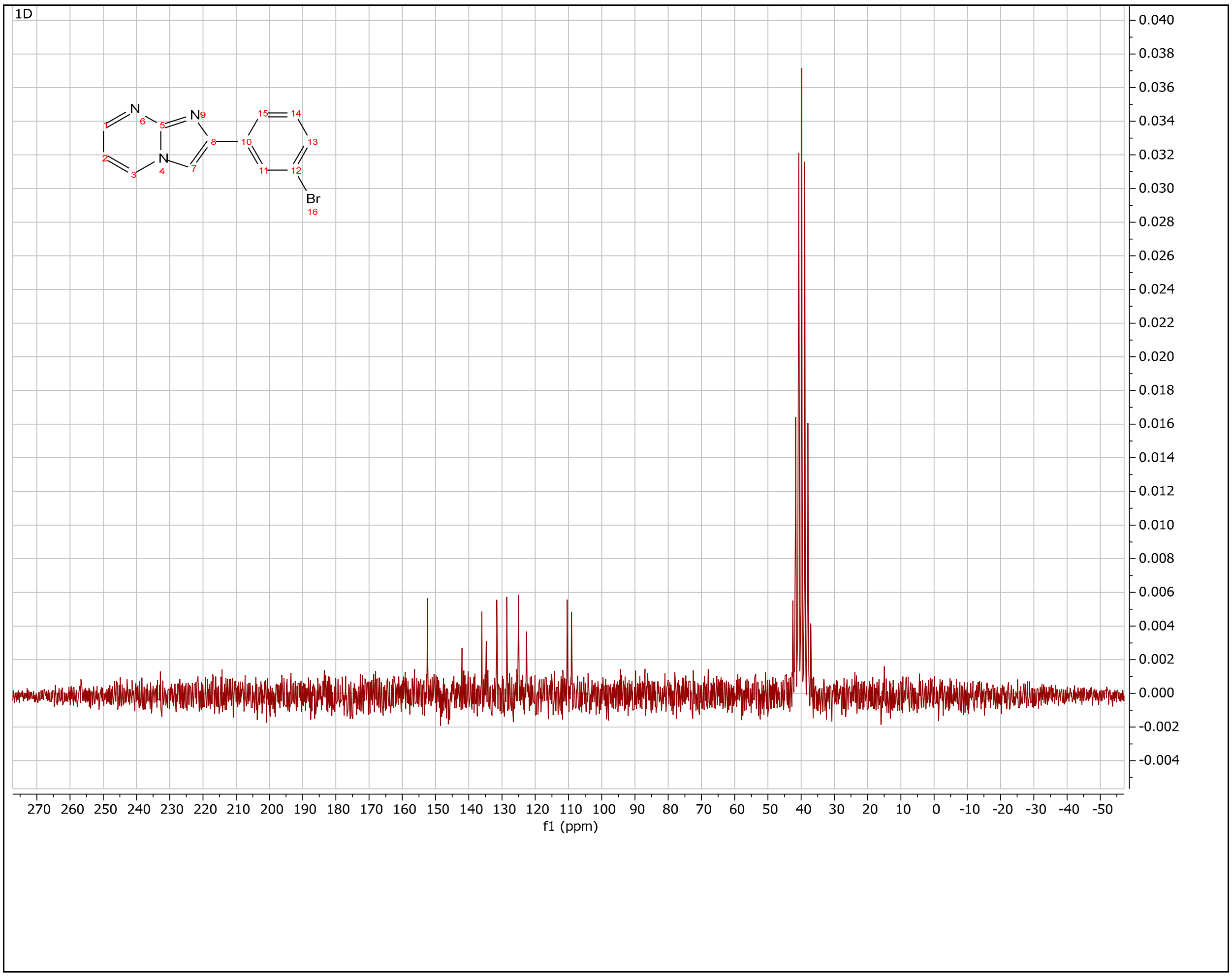
1D
f1 (ppm)
Br

Analysis Info

Sample Name	5 APCI BD13 274_43_01_962.d	Acquisition Date	08/03/2022 13:05:14
Method	962.m	Instrument	amaZon ETD

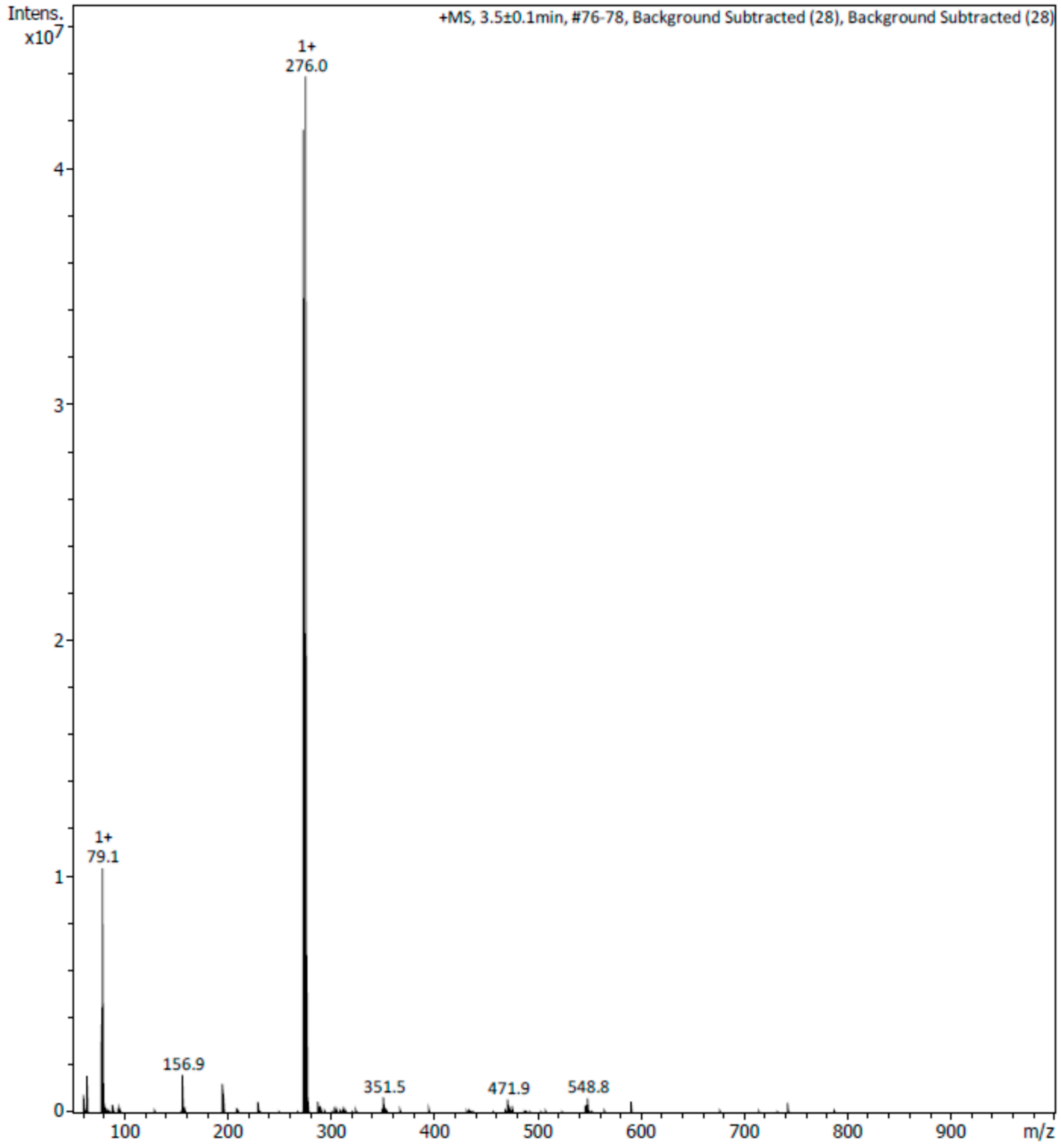

3c

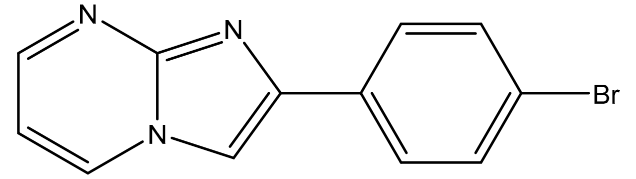
N
N
N
Br

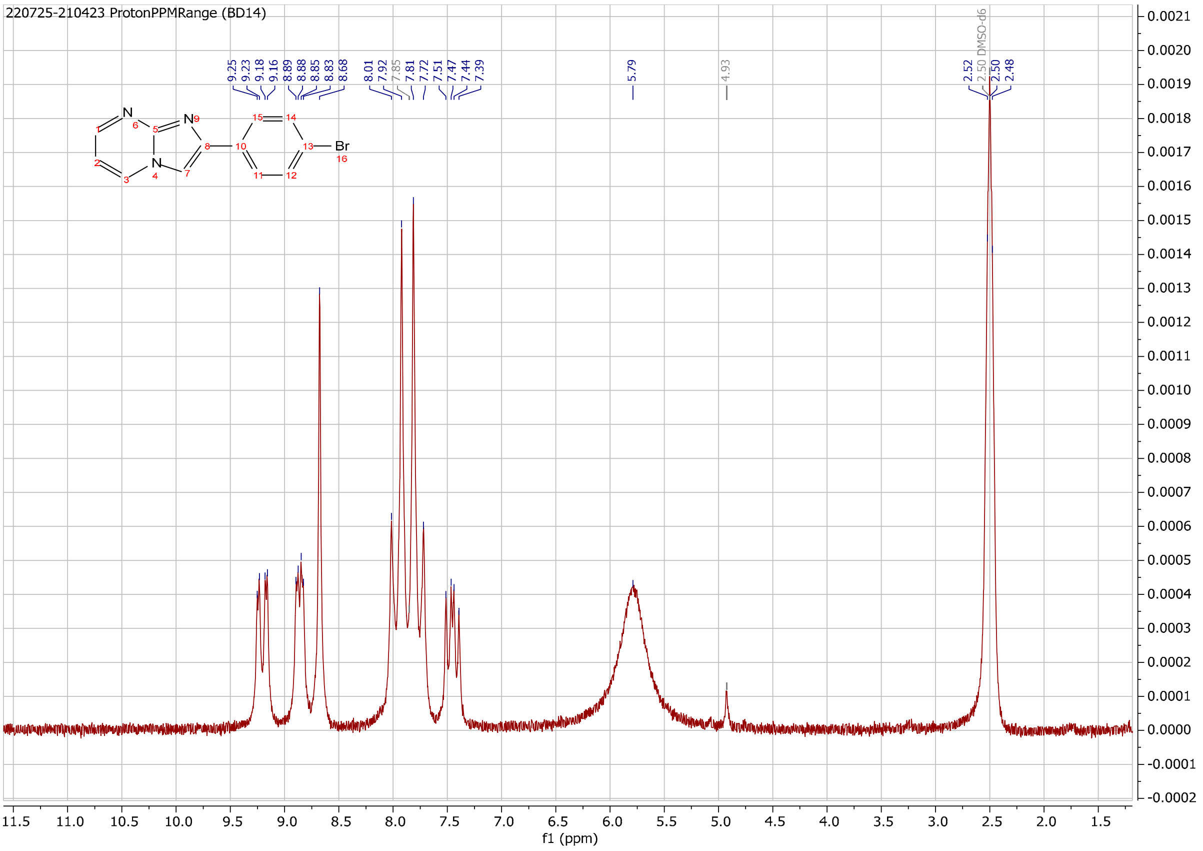
220725-210423 ProtonPPMRange (BD14)
9.25
9.23
9.18
9.16
8.89
8.88
8.85
8.83
8.68
8.01
7.92
7.85
7.81
7.72
7.51
7.47
7.44
7.39
5.79
4.93
2.52
2.50 DMSO-d6
2.50
2.48
f1 (ppm)

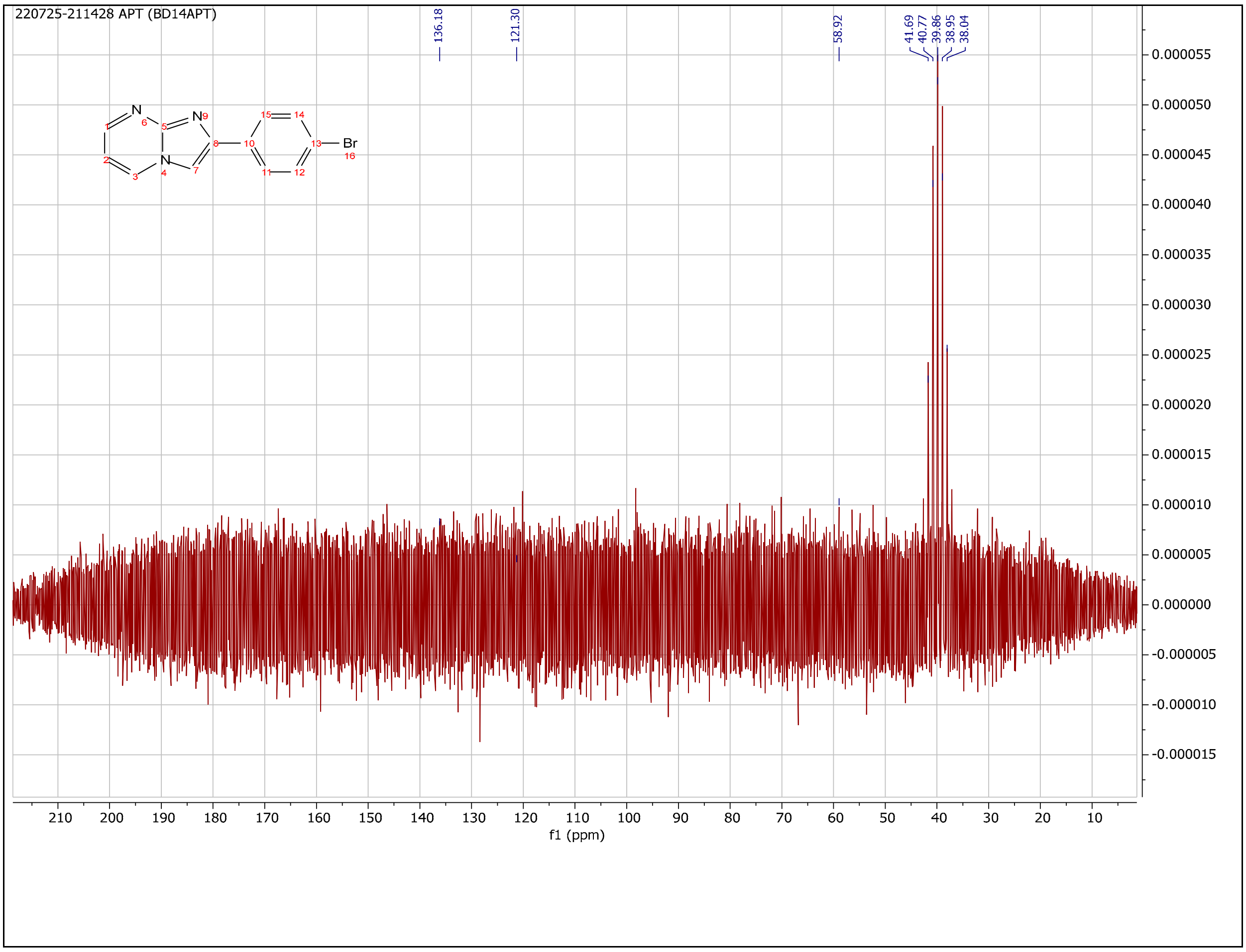
220725-211428 APT (BD14APT)
136.18
121.30
58.92
41.69
40.77
39.86
38.95
38.04
Br
f1 (ppm)

Analysis Info

Sample Name	4 APCI BD14 274_42_01_968.d	Acquisition Date	08/04/2022 11:04:16
Method	968.m	Instrument	amaZon ETD

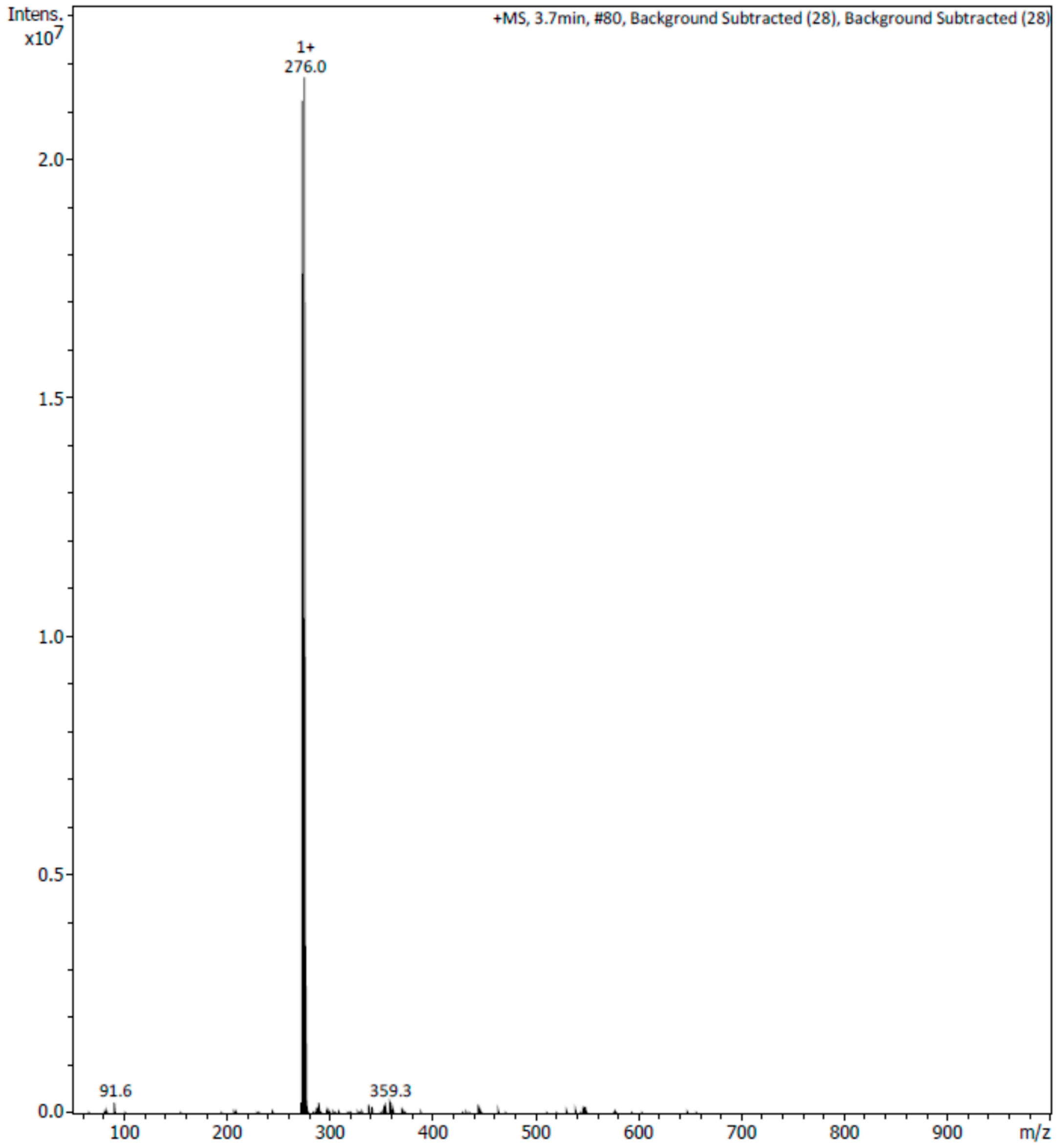

**3d**	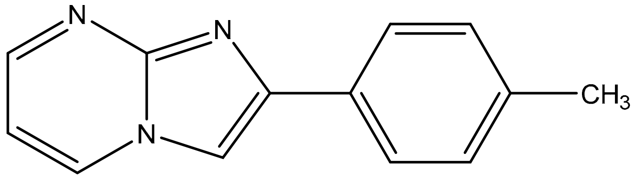

1D

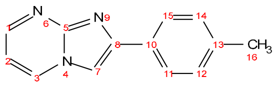

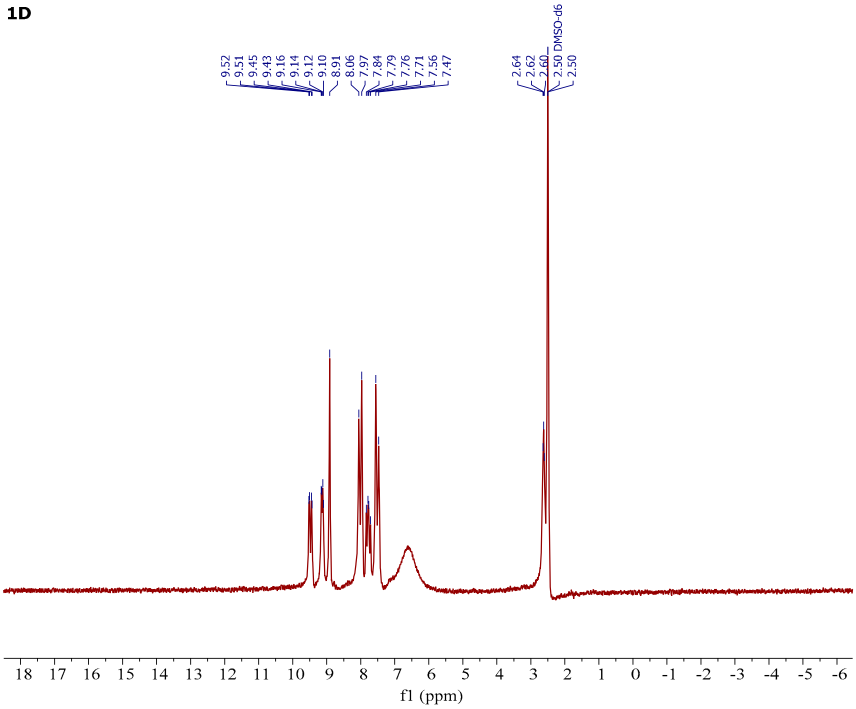

	Parámetro	Valor
1	Nombre del Archivo de Datos	c:/ projects/ data/ 2022/ 07/ 27/ Usuario/ 091843-BD15-COSY+/ pilotscan/ data.1d
2	Título	1D
3	Comentario	
4	Origen	
5	Propietario	
6	Sitio	
7	Instrumento	
8	Autor	
9	Disolvente	
10	Temperatura	
11	Secuencia del Pulso	1D
12	Experimento	1D
13	Sonda	
14	Número de Scans	1
15	Ganancia Recibida	22.0
16	Retardo de la Relajación	36.3698
17	Ancho del Pulso	13.4000
18	Frecuencia de	

1D

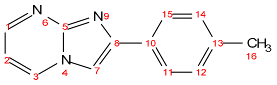

Parámetro	Valor
1 Nombre del Archivo de Datos	c:/ projects/ data/ 2022/ 07/ 26/ Usuario/ 114750-BD15-1D CARBON+ WALTZ/ data.1d
2 Título	1D
3 Comentario	
4 Origen	
5 Propietario	
6 Sitio	
7 Instrumento	
8 Autor	
9 Disolvente	
10 Temperatura	
11 Secuencia del Pulso	1D
12 Experimento	1D
13 Sonda	
14 Número de Scans	2361

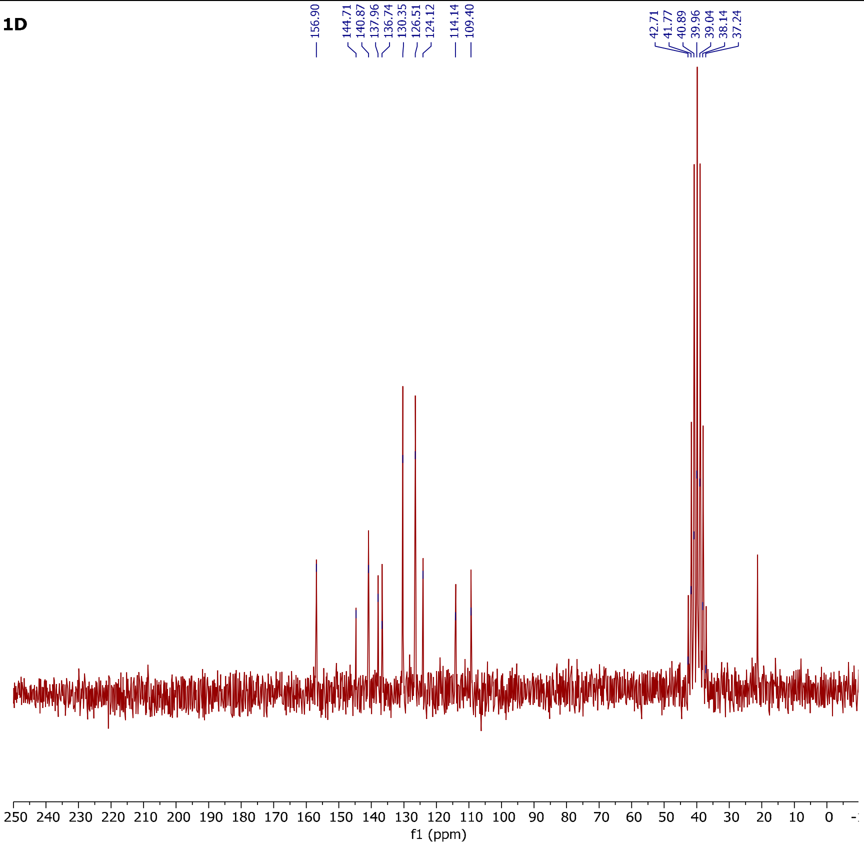

Analysis Info

Sample Name	5 APCI BD15 209_43_01_969.d	Acquisition Date	08/04/2022 11:15:21
Method	969.m	Instrument	amaZon ETD

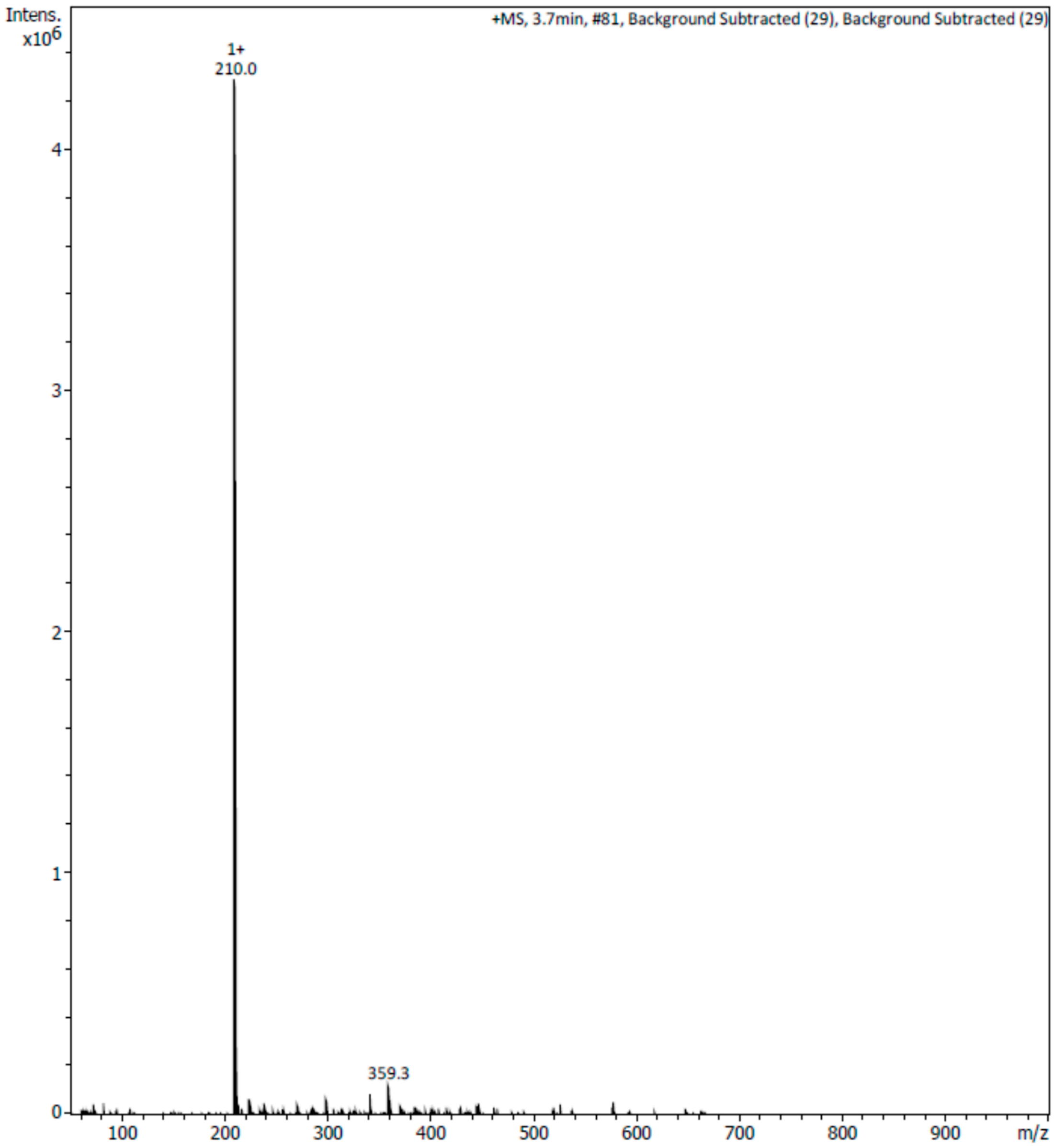

**3e**	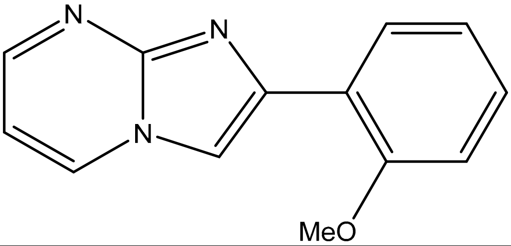

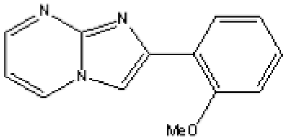

	Parámetro	Valor
1	Nombre del Archivo de Datos	c:/ projects/ data/ 2022/ 07/ 27/ Usuario/ 101945-BD16-1D EXTENDED+/ data. 1d
2	Título	1D
3	Comentario	
4	Origen	
5	Propietario	
6	Sitio	
7	Instrumento	
8	Autor	
9	Disolvente	
10	Temperatura	
11	Secuencia del Pulso	1D
12	Experimento	1D
13	Sonda	
14	Número de Scans	18
15	Ganancia Recibida	22.0
16	Retardo de la Relajación	36.3698
17	Ancho del Pulso	13.4000

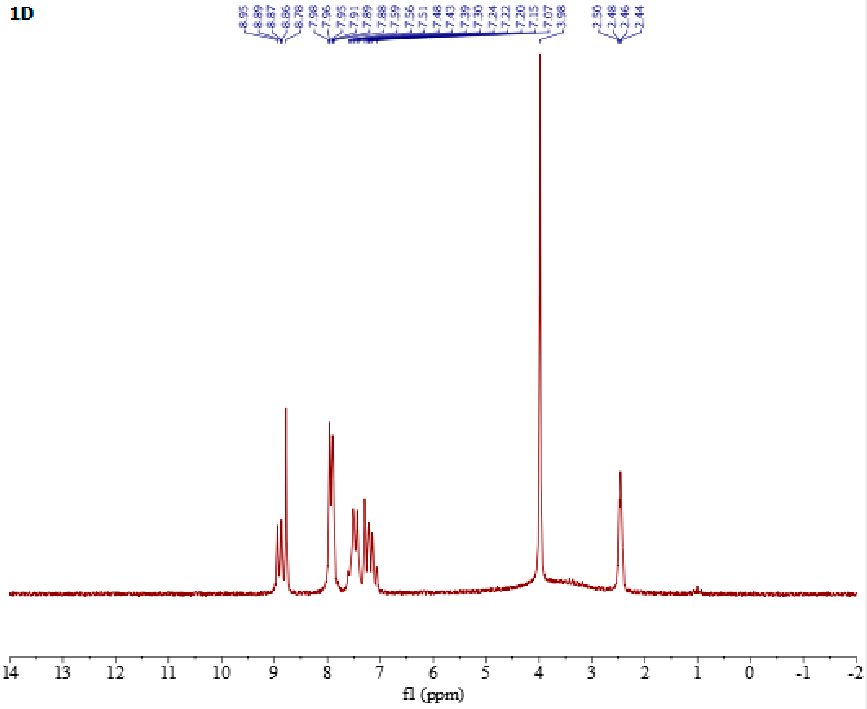

magritek

1D

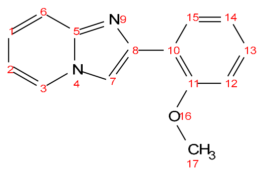

	Parámetro	Valor
1	Nombre del Archivo de Datos	c:/ projects/ data/ 2022/ 07/ 27/ Usuario/ 104431-BD16-1D CARBON+ WALTZ/ data.1d
2	Título	1D
3	Comentario	
4	Origen	
5	Propietario	
6	Sitio	
7	Instrumento	
8	Autor	
9	Disolvente	
10	Temperatura	
11	Secuencia del Pulso	1D
12	Experimento	1D
13	Sonda	
14	Número de Scans	487

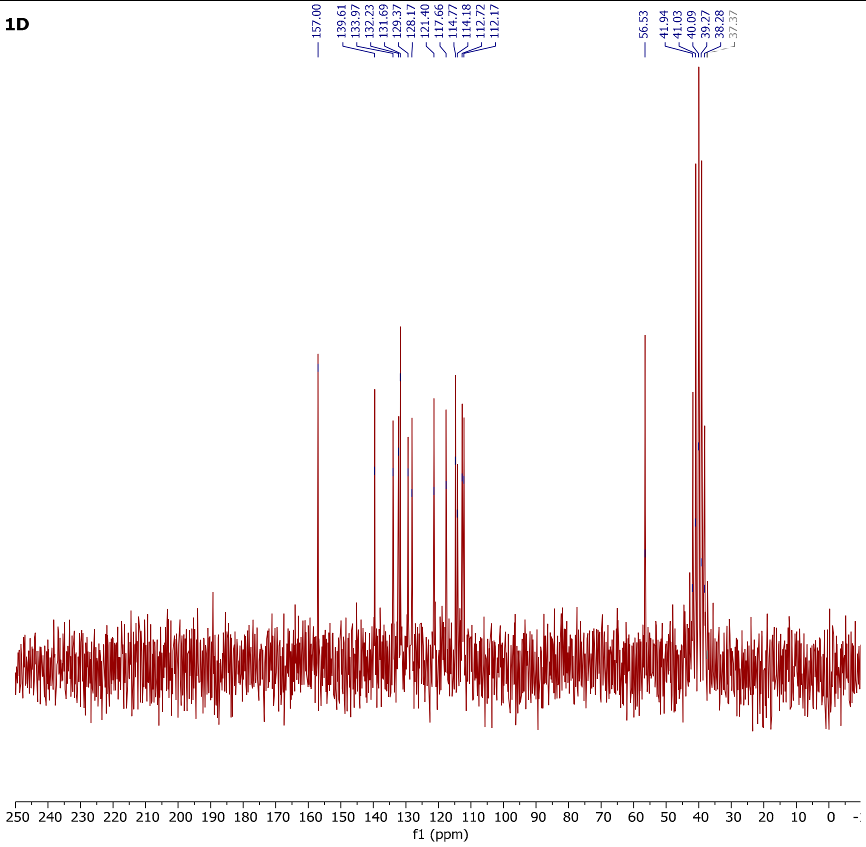

Analysis Info

Sample Name	6 APCI BD16 225_44_01_963.d	Acquisition Date	08/03/2022 13:16:21
Method	963.m	Instrument	amaZon ETD

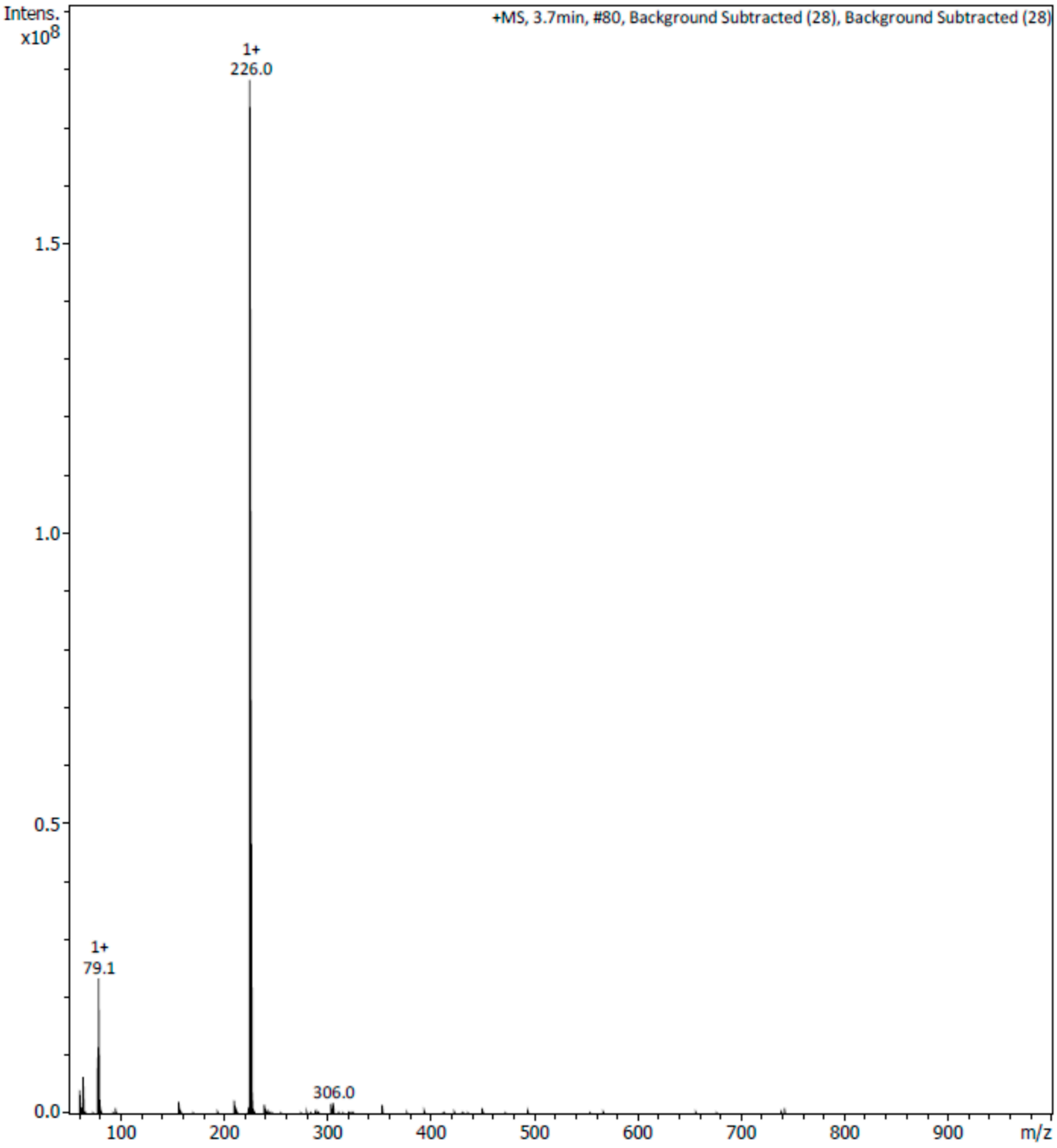

3f

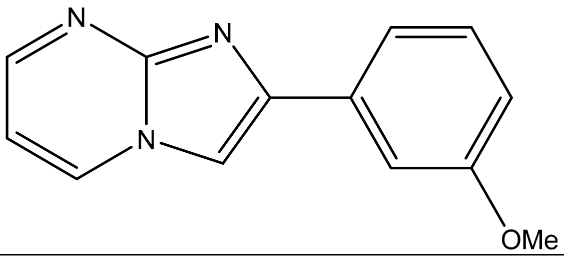
N
N
N
OMe

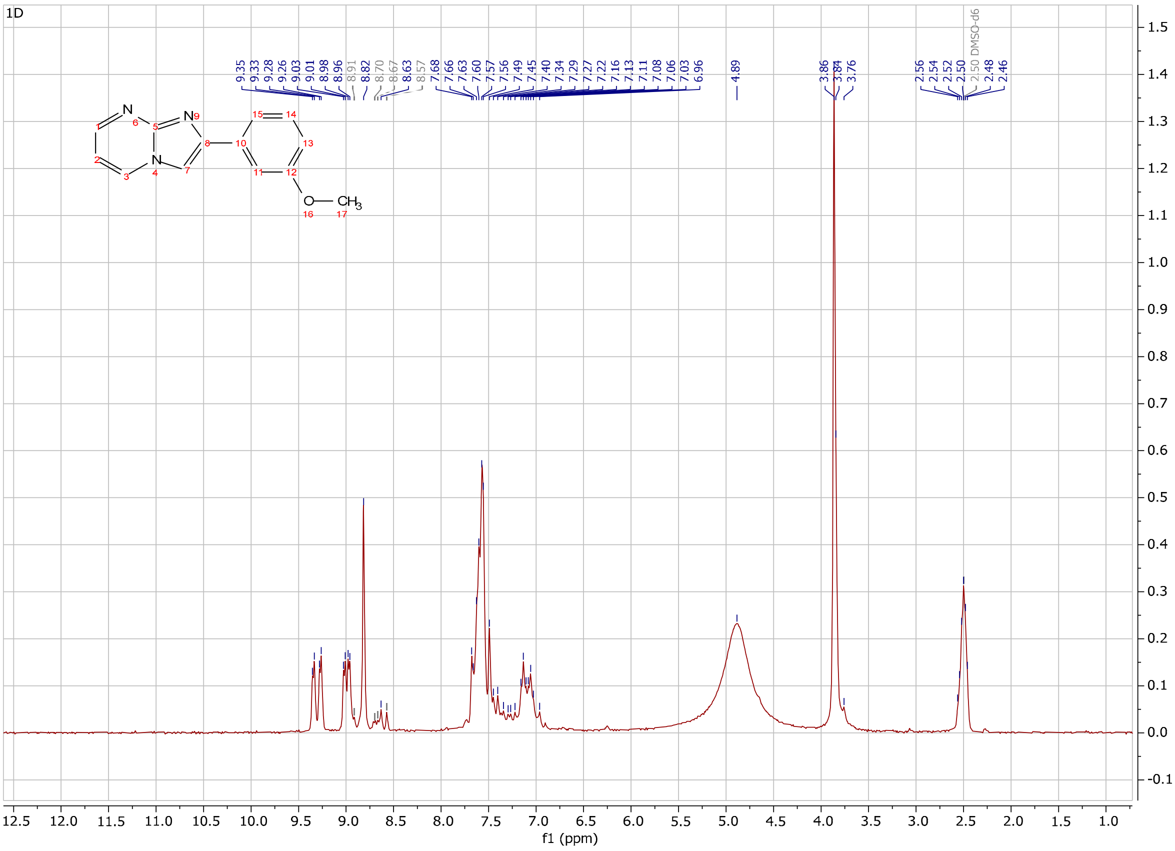
1D
9.35
9.33
9.28
9.26
9.03
9.01
8.98
8.96
8.91
8.82
8.70
8.67
8.63
8.57
7.68
7.66
7.63
7.60
7.57
7.56
7.49
7.45
7.40
7.34
7.29
7.27
7.22
7.16
7.13
7.11
7.08
7.06
7.03
6.96
4.89
3.86
3.84
3.76
2.56
2.54
2.52
2.50 DMSO-d6
2.48
2.46
f1 (ppm)

1D

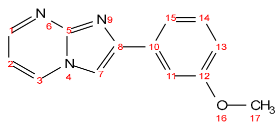

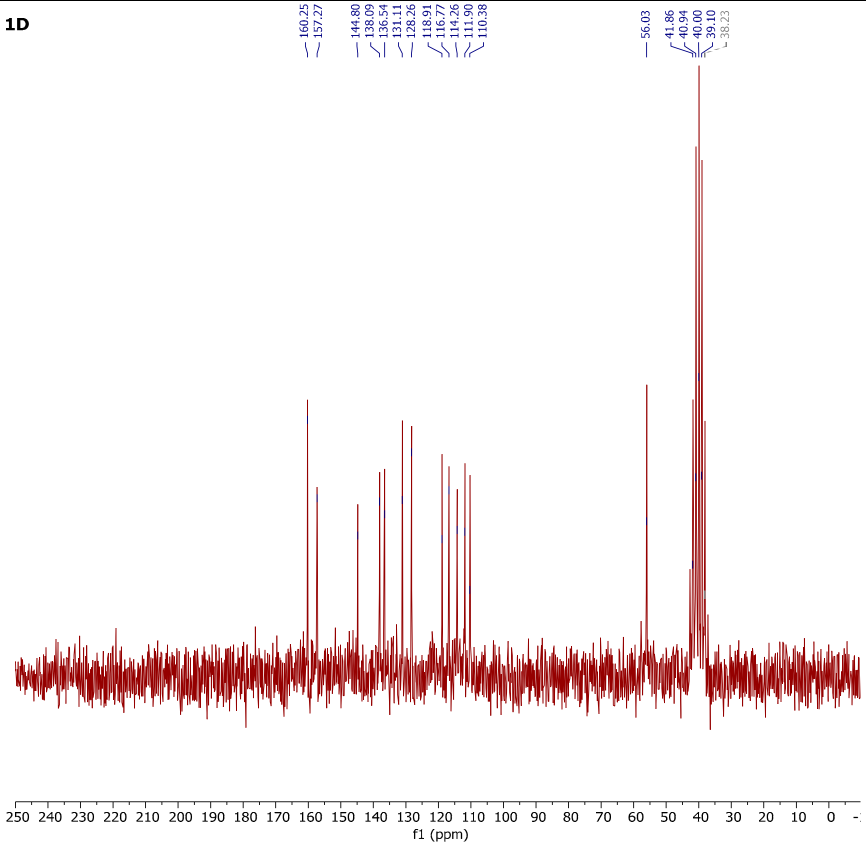

	Parámetro	Valor
1	Nombre del Archivo de Datos	c:/ projects/ data/ 2022/ 07/ 27/ Usuario/ 135259-BD16-1D CARBON WALTZ/ data.1d
2	Título	1D
3	Comentario	
4	Origen	
5	Propietario	
6	Sitio	
7	Instrumento	
8	Autor	
9	Disolvente	
10	Temperatura	
11	Secuencia del Pulso	1D
12	Experimento	1D
13	Sonda	
14	Número de Scans	1514

Analysis Info

Sample Name	6 APCI BD17 225_44_01_970.d	Acquisition Date	08/04/2022 11:26:27
Method	970.m	Instrument	amaZon ETD

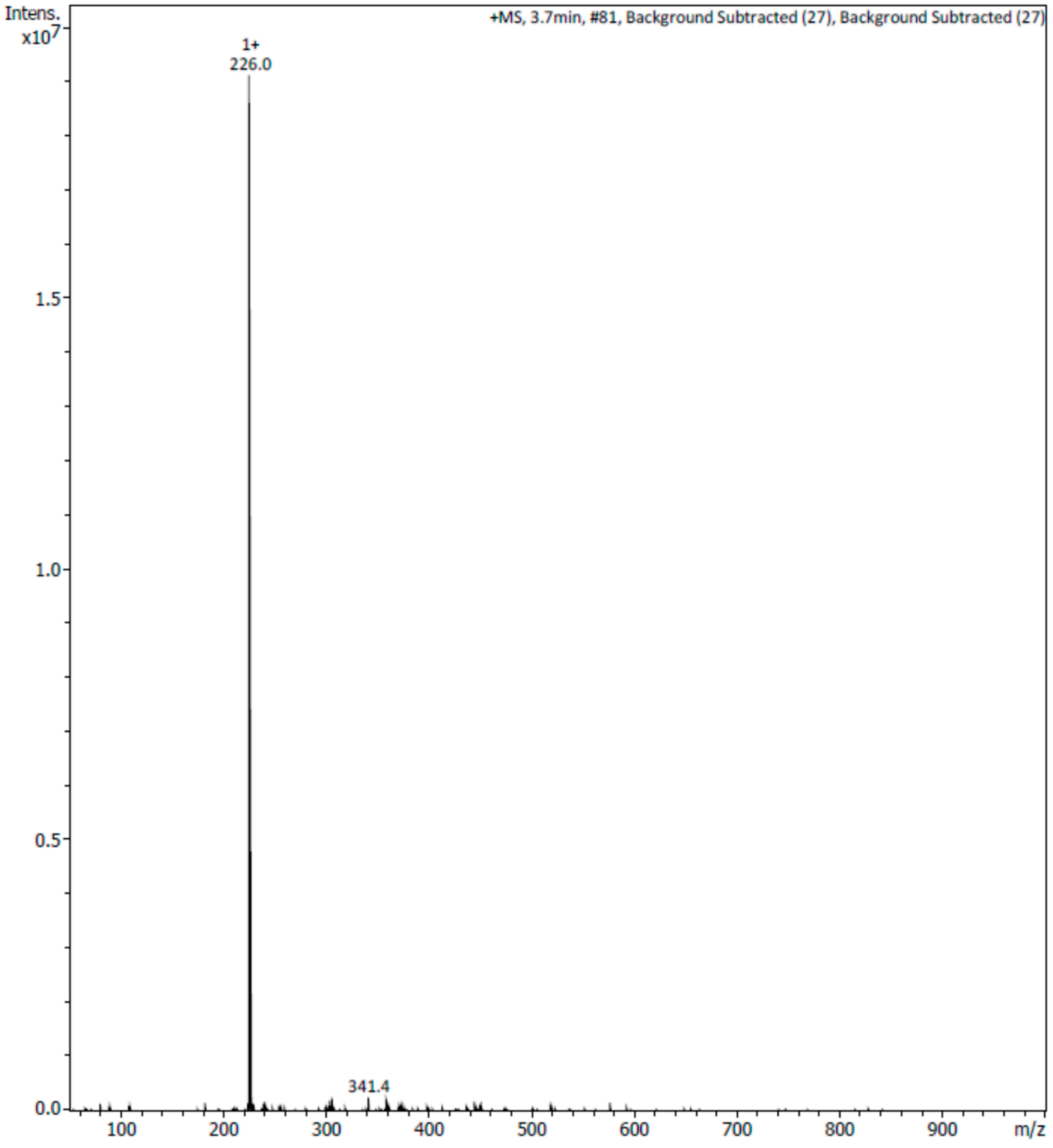

3g

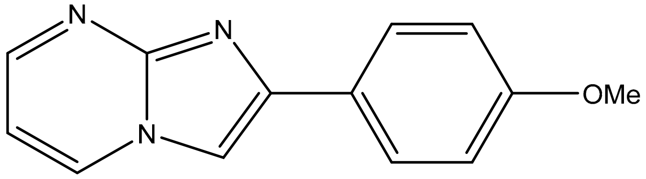

N
N
N
OMe

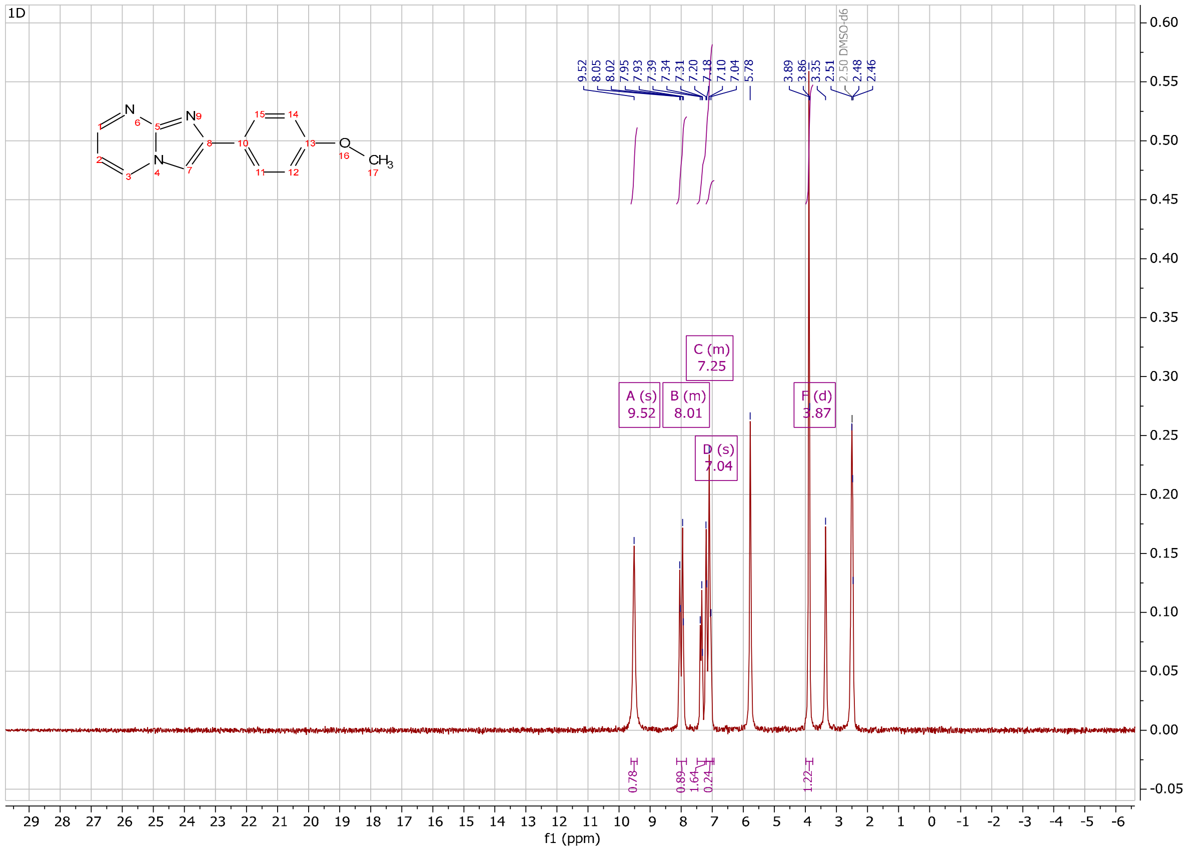

1D
9.52
8.05
8.02
7.95
7.93
7.39
7.34
7.31
7.20
7.18
7.10
7.04
5.78
3.89
3.86
3.35
2.51
2.50 DMSO-d6
2.48
2.46
A (s)
9.52
B (m)
8.01
C (m)
7.25
D (s)
7.04
F (d)
3.87
0.78
0.89
1.64
0.24
1.22
f1 (ppm)

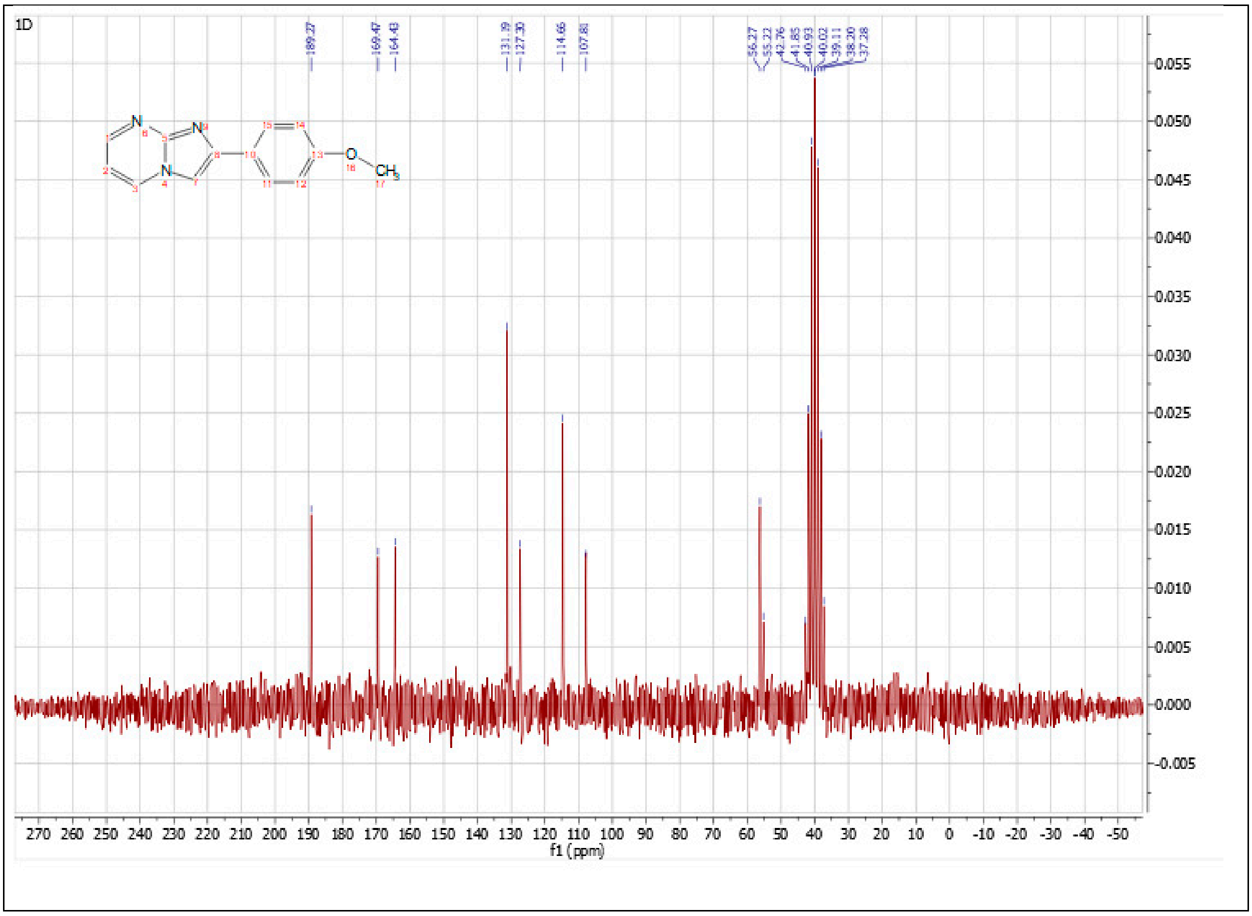
1D
189.27
169.47
164.43
131.19
127.30
114.66
107.81
56.27
55.22
42.76
41.85
40.93
40.02
39.11
38.20
37.28
f1 (ppm)

Analysis Info

Sample Name	8 APCI BD18 225_45_01_972.d	Acquisition Date	08/04/2022 11:48:35
Method	972.m	Instrument	amaZon ETD

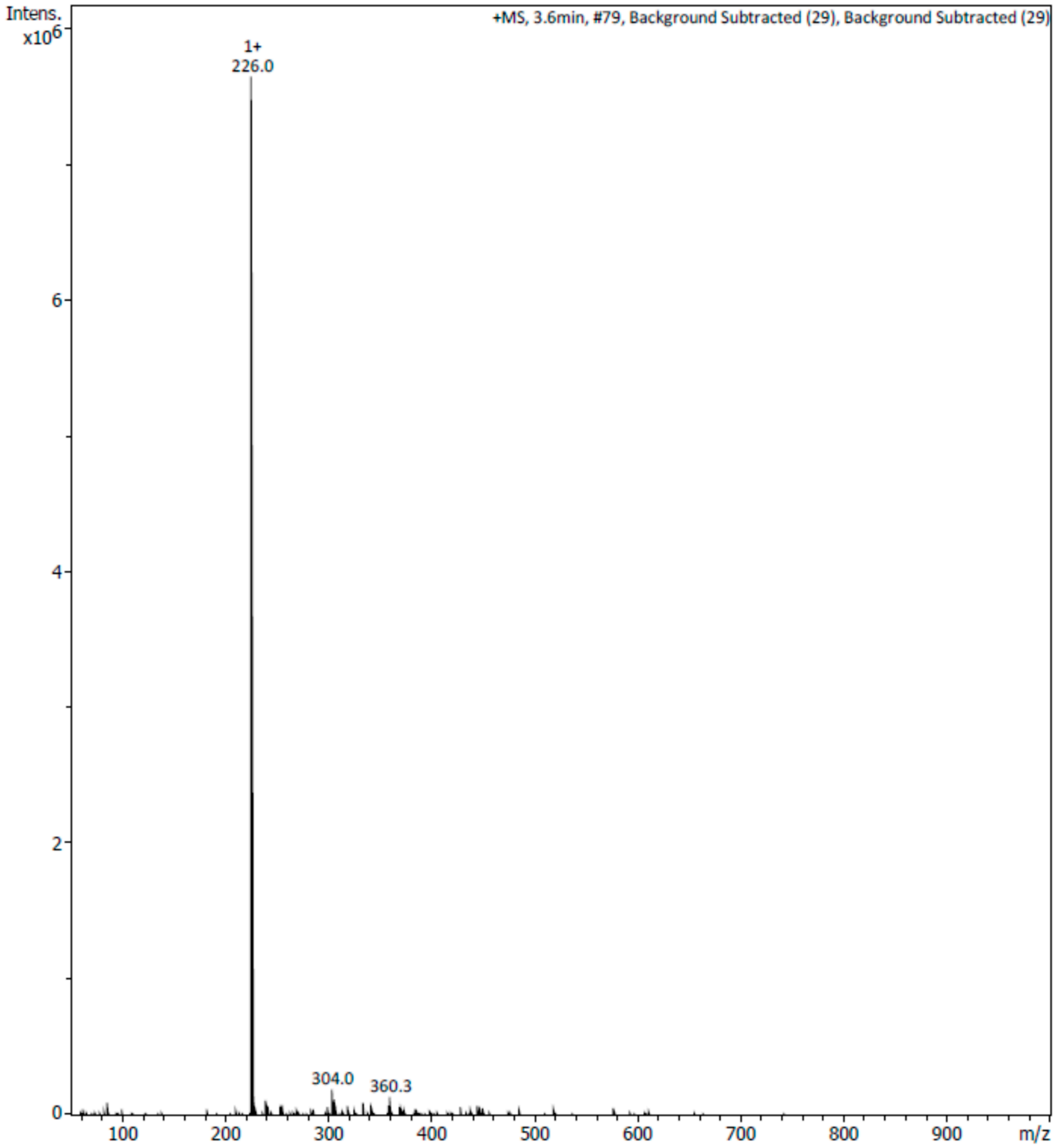

**3h**	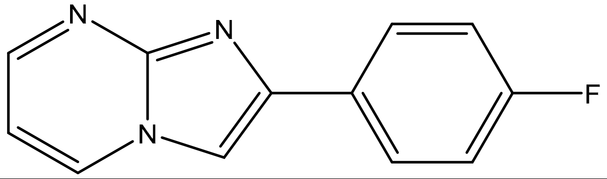

magritek

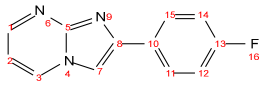

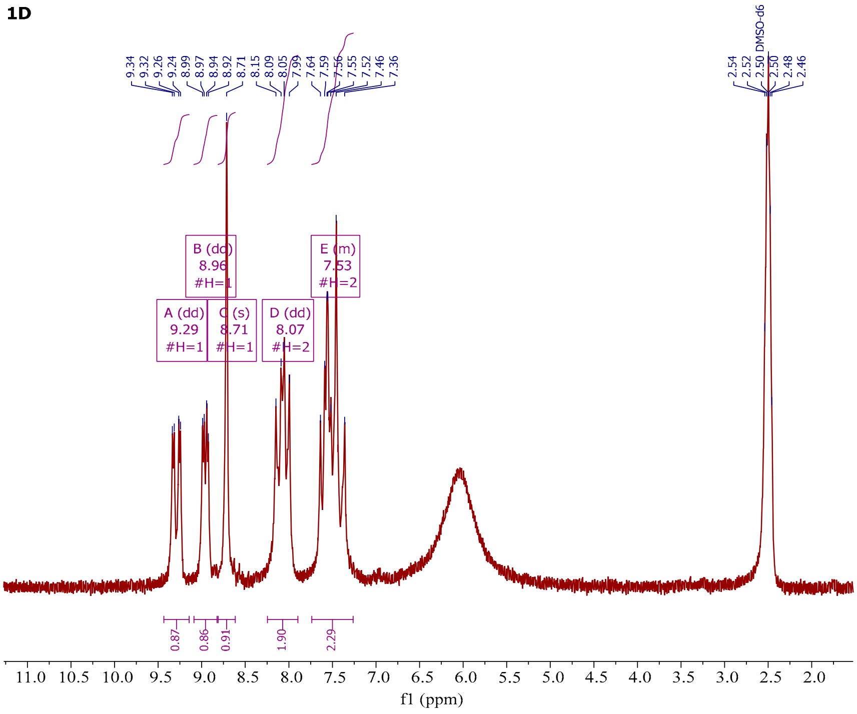

	Parámetro	Valor
1	Nombre del Archivo de Datos	c:/ projects/ data/ 2022/ 07/ 28/ Usuario/ 092505-BD19-1D EXTENDED+/ data.1d
2	Título	1D
3	Comentario	
4	Origen	
5	Propietario	
6	Sitio	
7	Instrumento	
8	Autor	
9	Disolvente	
10	Temperatura	
11	Secuencia del Pulso	1D
12	Experimento	1D
13	Sonda	
14	Número de Scans	33
15	Ganancia Recibida	22.0

magritek

1D

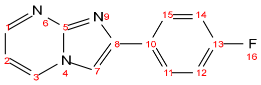

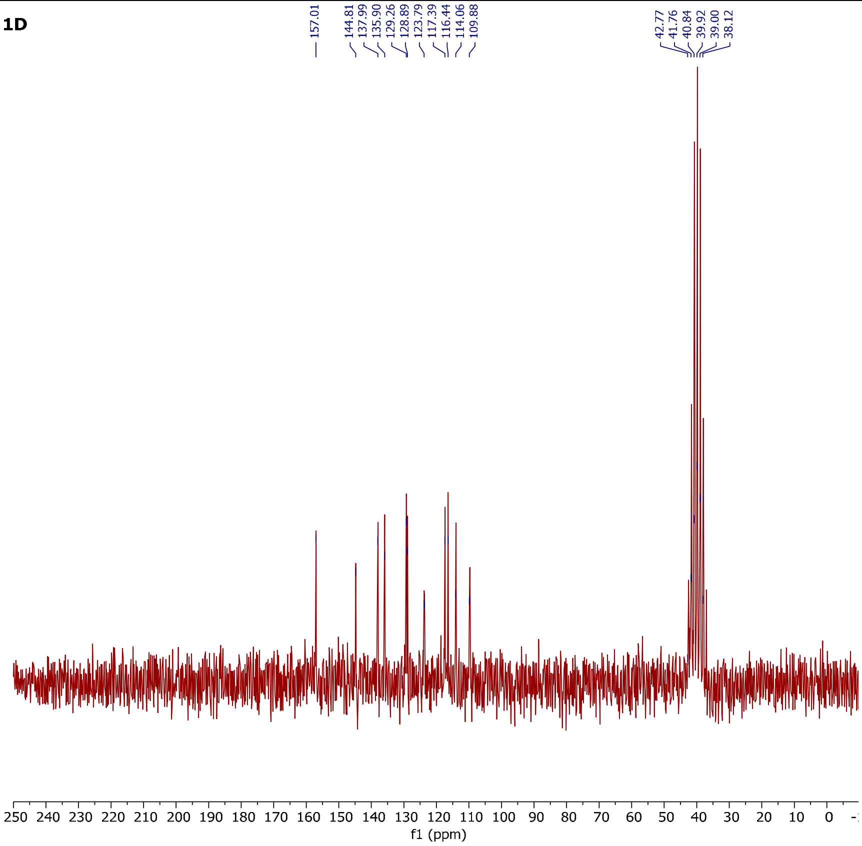

	Parámetro	Valor
1	Nombre del Archivo de Datos	c:/ projects/ data/ 2022/ 07/ 28/ Usuario/ 095229-BD19-1D CARBON+ WALTZ/ data.1d
2	Título	1D
3	Comentario	
4	Origen	
5	Propietario	
6	Sitio	
7	Instrumento	
8	Autor	
9	Disolvente	
10	Temperatura	
11	Secuencia del Pulso	1D
12	Experimento	1D
13	Sonda	
14	Número de Scans	1062

Analysis Info

Sample Name	9 APCI BD19 213_46_01_973.d	Acquisition Date	08/04/2022 11:59:40
Method	973.m	Instrument	amaZon ETD

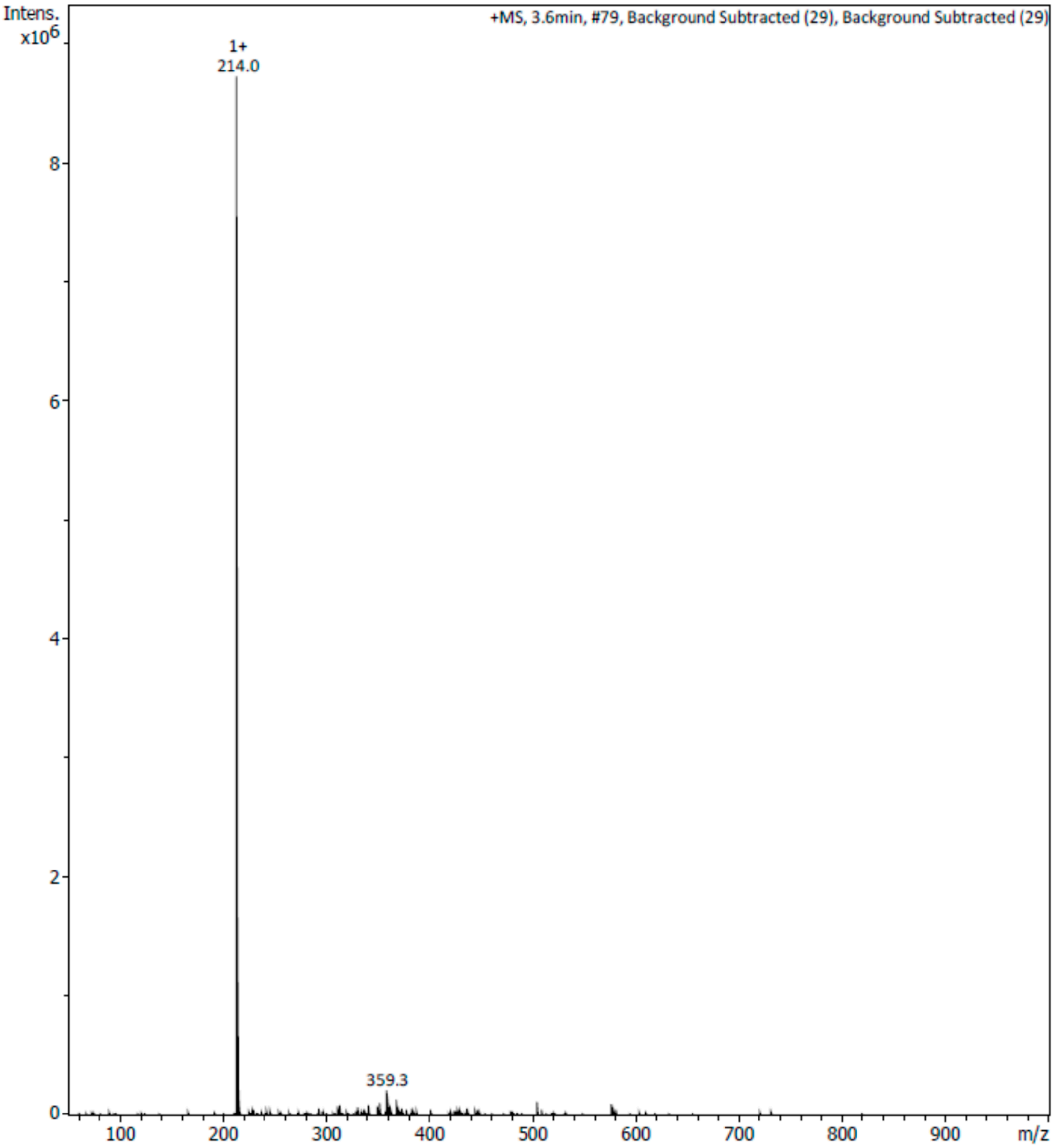

3i

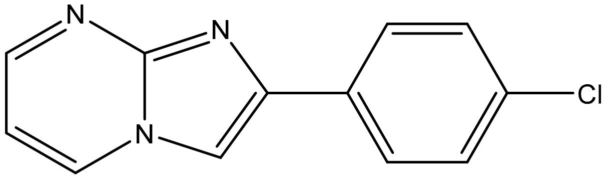

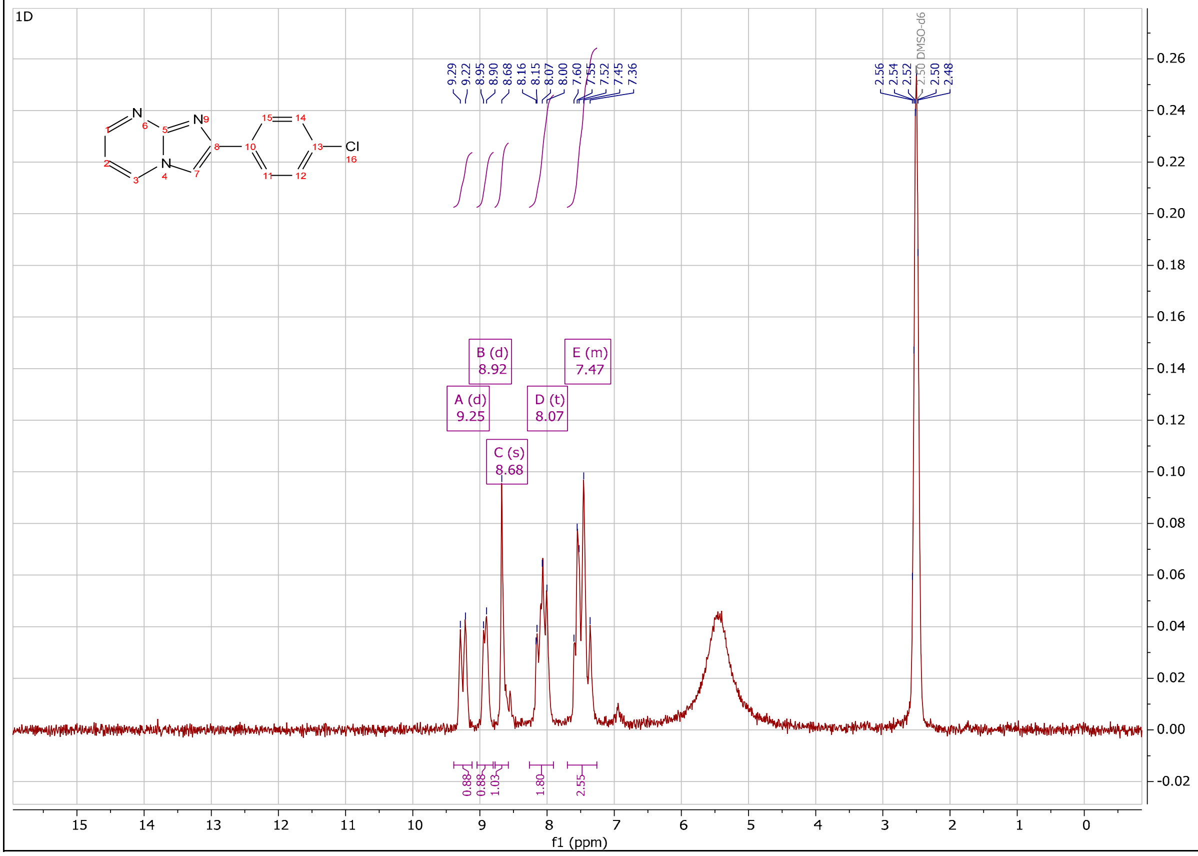

1D
9.29
9.22
8.95
8.90
8.68
8.16
8.15
8.07
8.00
7.60
7.55
7.52
7.45
7.36
2.56
2.54
2.52
2.50 DMSO-d6
2.50
2.48
A (d)
9.25
B (d)
8.92
C (s)
8.68
D (t)
8.07
E (m)
7.47
0.88
0.88
1.03
1.80
2.55
f1 (ppm)

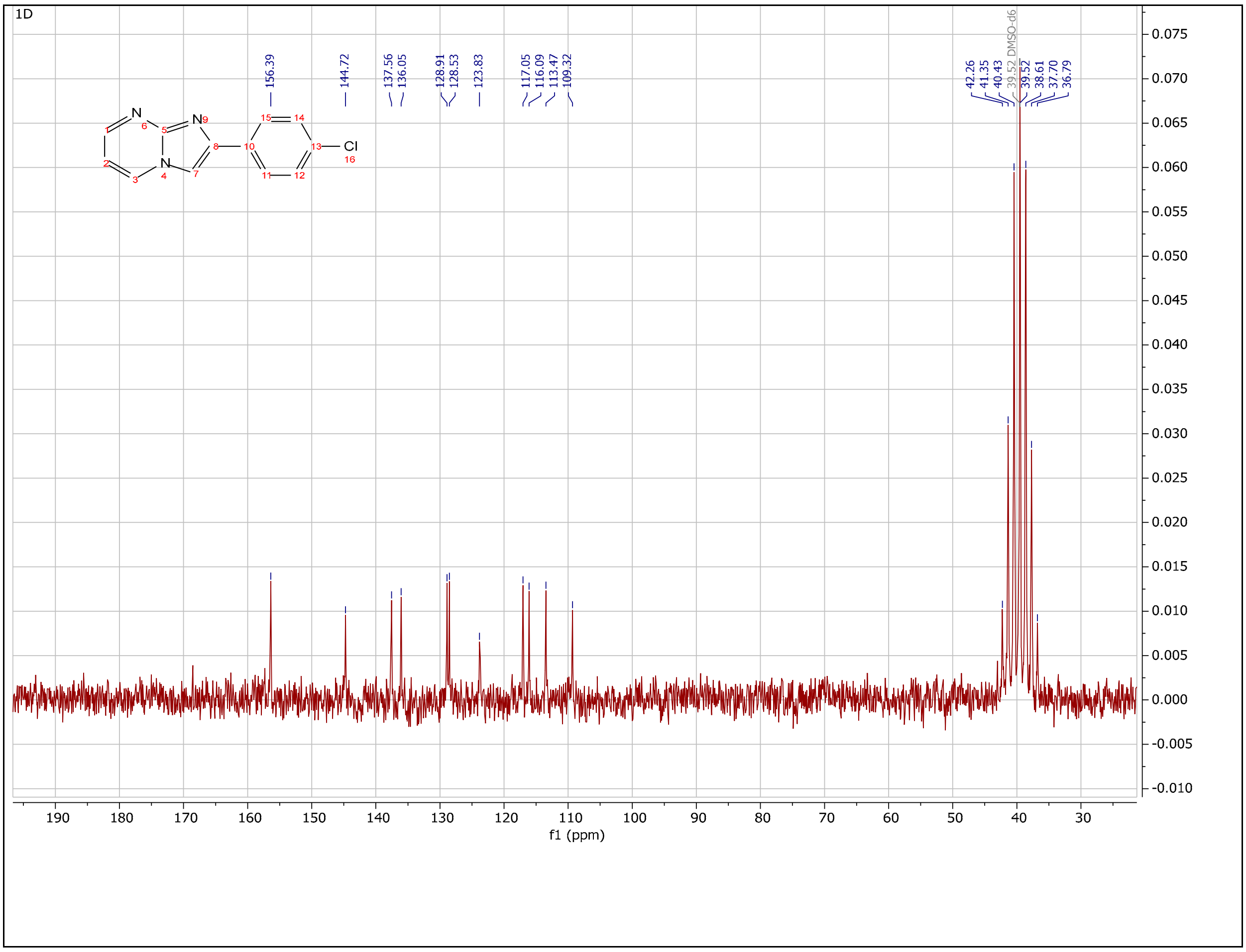
1D
156.39
144.72
137.56
136.05
128.91
128.53
123.83
117.05
116.09
113.47
109.32
42.26
41.35
40.43
39.52 DMSO-d6
39.52
38.61
37.70
36.79
N
N
N
Cl
f1 (ppm)

Analysis Info

Sample Name	3 APCI BD20 229_61_01_993.d	Acquisition Date	05/08/2022 11:07:56
Method	993.m	Instrument	amaZon ETD

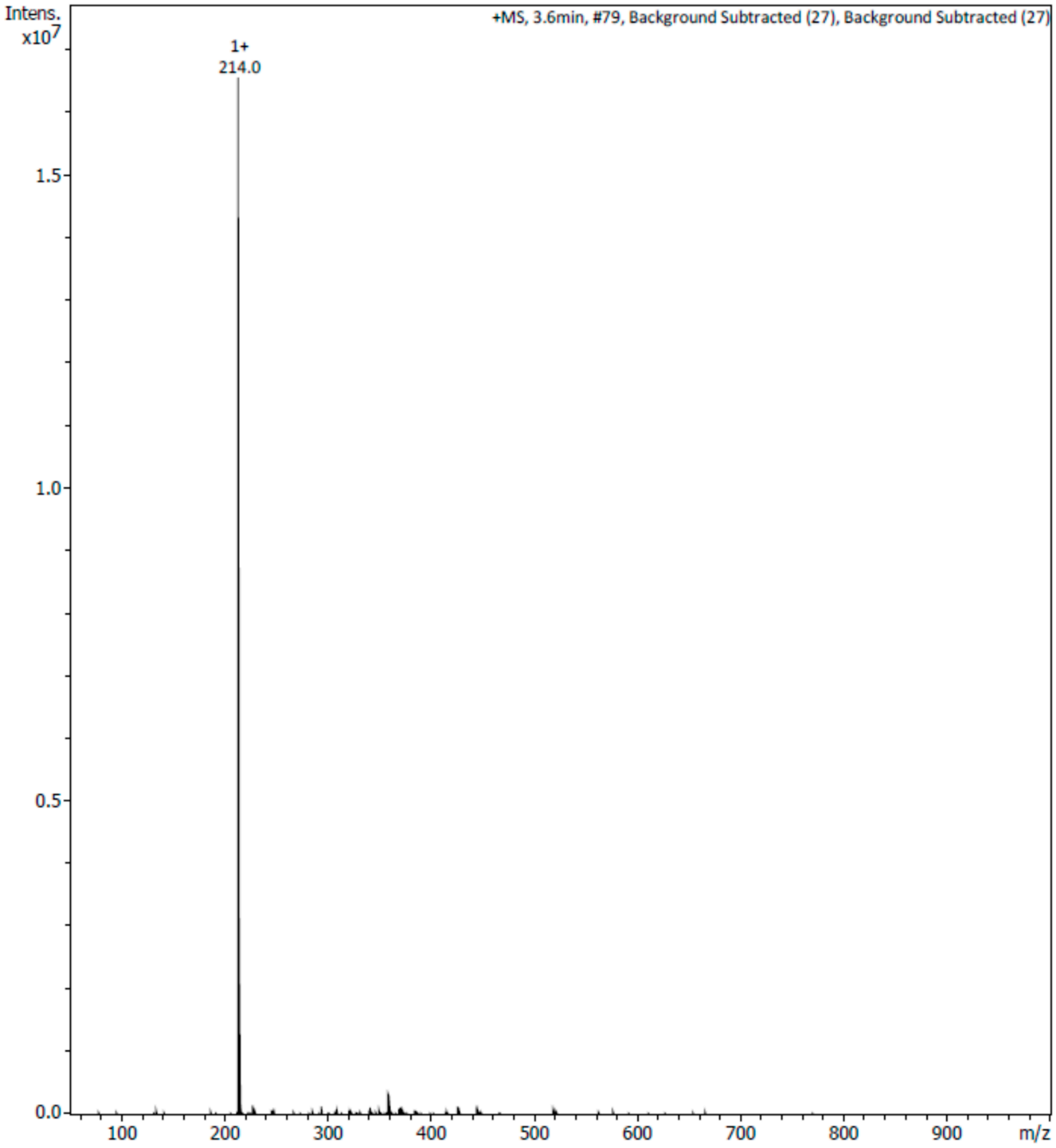

**3j**	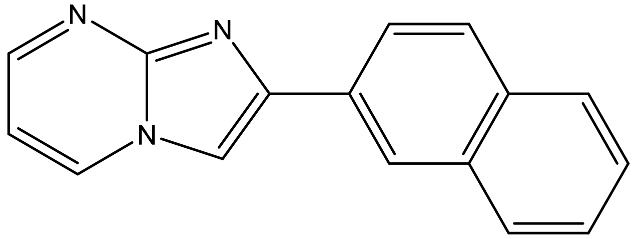

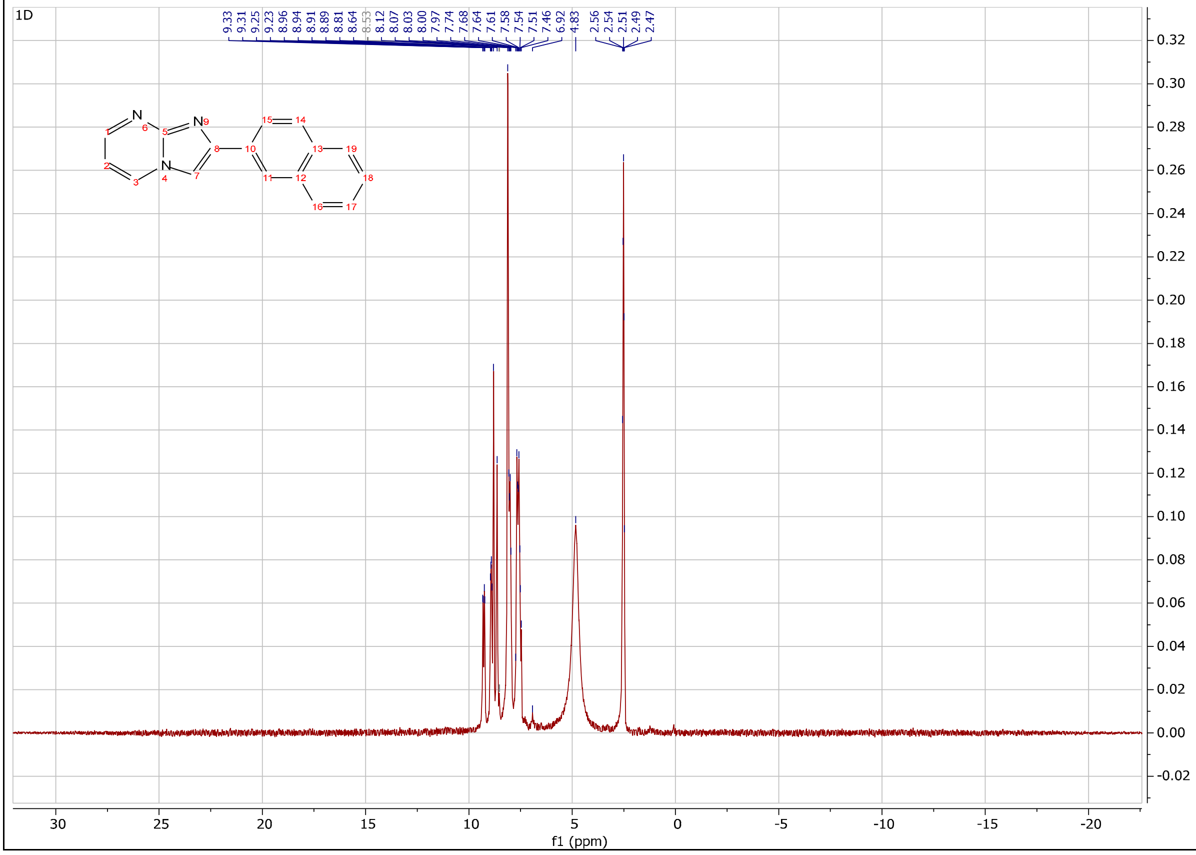
1D
9.33 9.31 9.25 9.23 8.96 8.94 8.91 8.89 8.81 8.64 8.53 8.12 8.07 8.03 8.00 7.97 7.74 7.68 7.64 7.61 7.58 7.54 7.51 7.46 6.92 4.83 2.56 2.54 2.51 2.49 2.47
f1 (ppm)

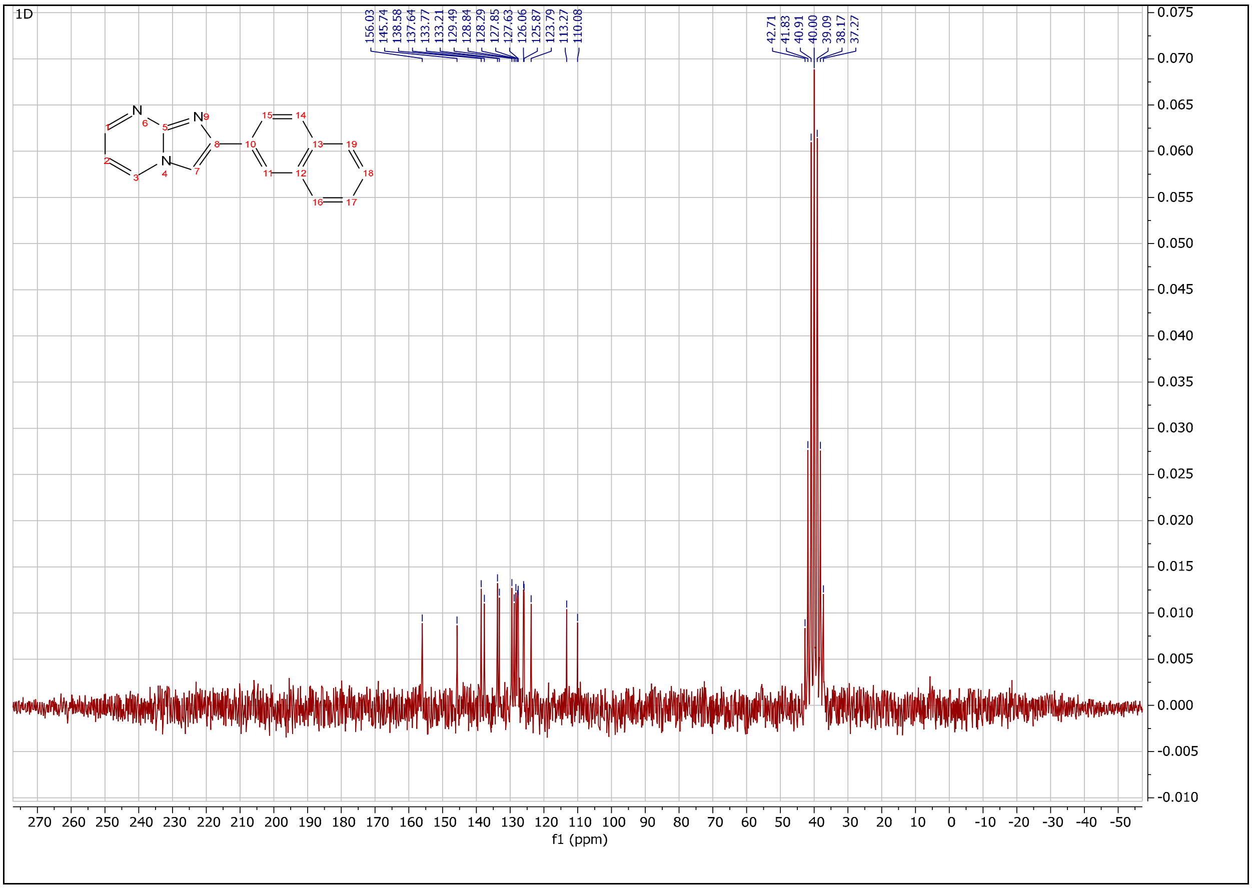

1D
156.03
145.74
138.58
137.64
133.77
133.21
129.49
128.84
128.29
127.85
127.63
126.06
125.87
123.79
113.27
110.08
42.71
41.83
40.91
40.00
39.09
38.17
37.27
f1 (ppm)

Analysis Info

Sample Name	11 APCI BD21 245_48_01_975.d	Acquisition Date	08/04/2022 12:21:51
Method	975.m	Instrument	amaZon ETD

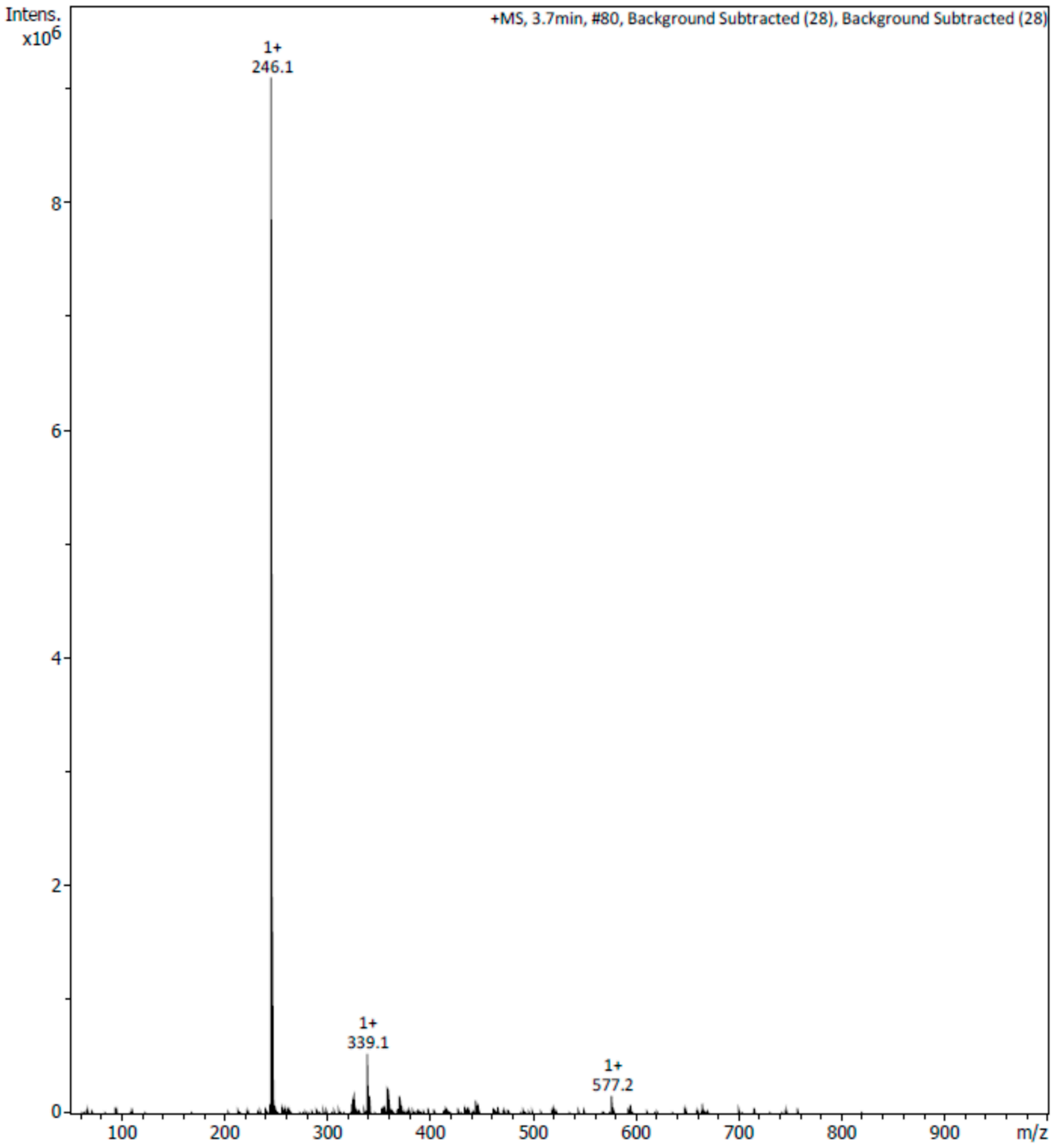

3k

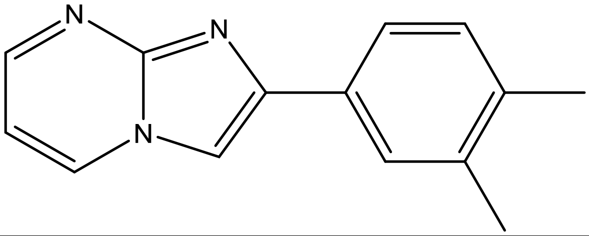

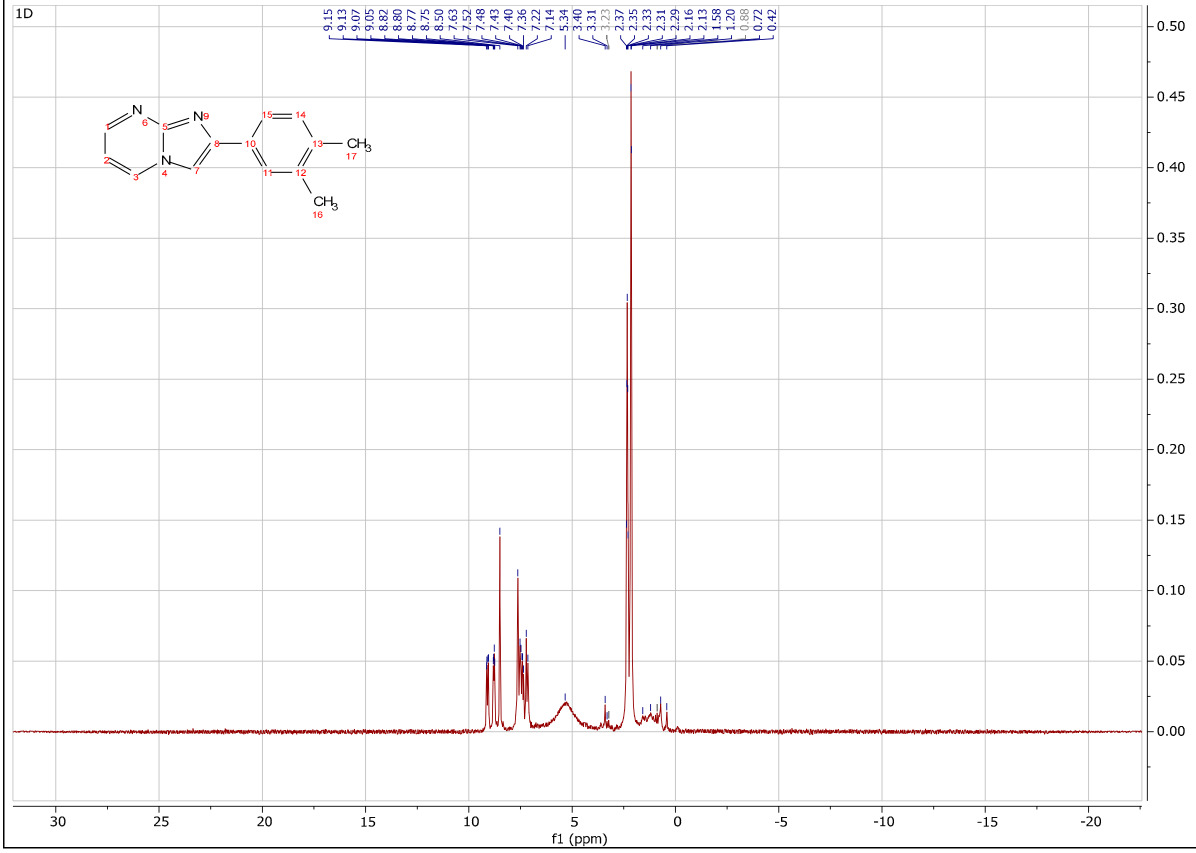
1D
9.15
9.13
9.07
9.05
8.82
8.80
8.77
8.75
8.50
7.63
7.52
7.48
7.43
7.40
7.36
7.22
7.14
5.34
3.40
3.31
3.23
2.37
2.35
2.33
2.31
2.29
2.16
2.13
1.58
1.20
0.88
0.72
0.42
f1 (ppm)

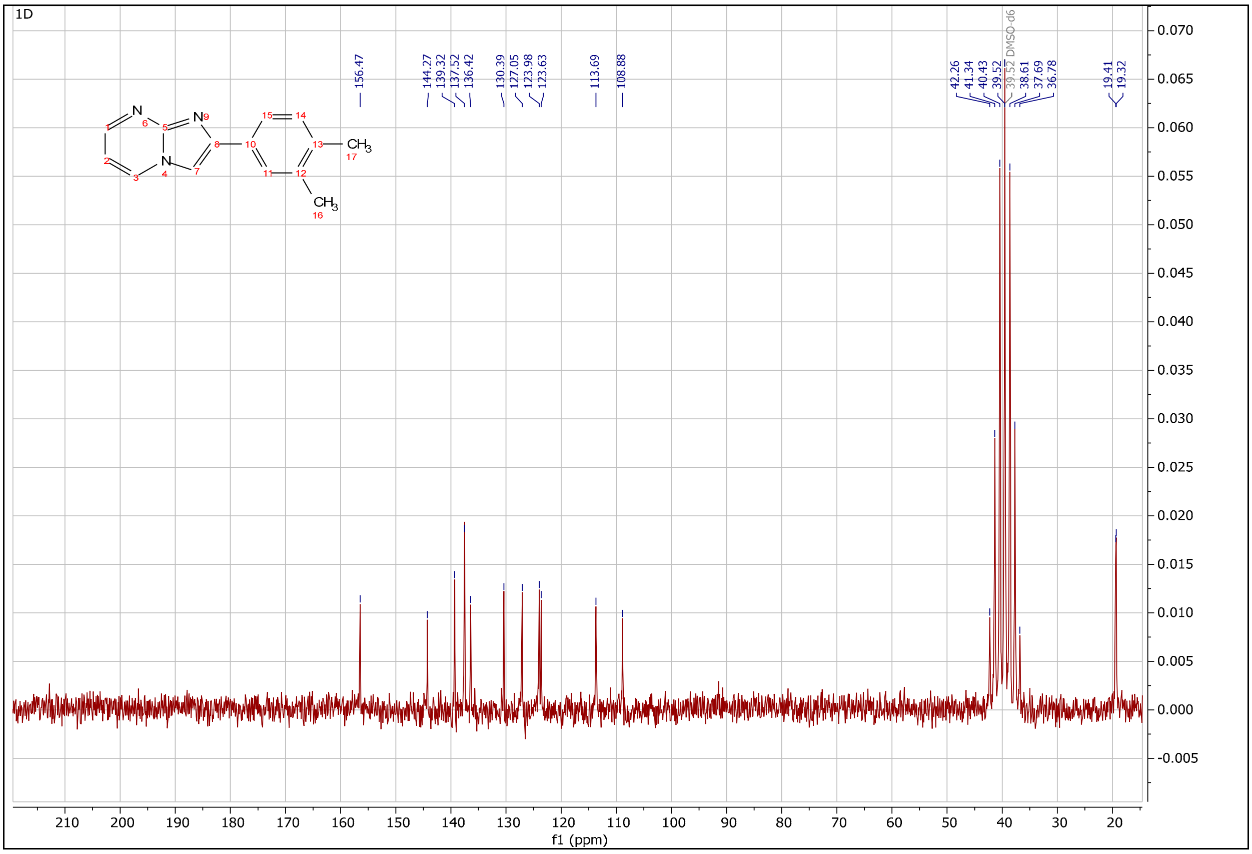
1D
156.47
144.27
139.32
137.52
136.42
130.39
127.05
123.98
123.63
113.69
108.88
42.26
41.34
40.43
39.52
39.52 DMSO-d6
38.61
37.69
36.78
19.41
19.32
CH3
CH3
f1 (ppm)

Analysis Info

Sample Name	12 APCI BD22 223_49_01_976.d	Acquisition Date	08/04/2022 12:32:58
Method	976.m	Instrument	amaZon ETD

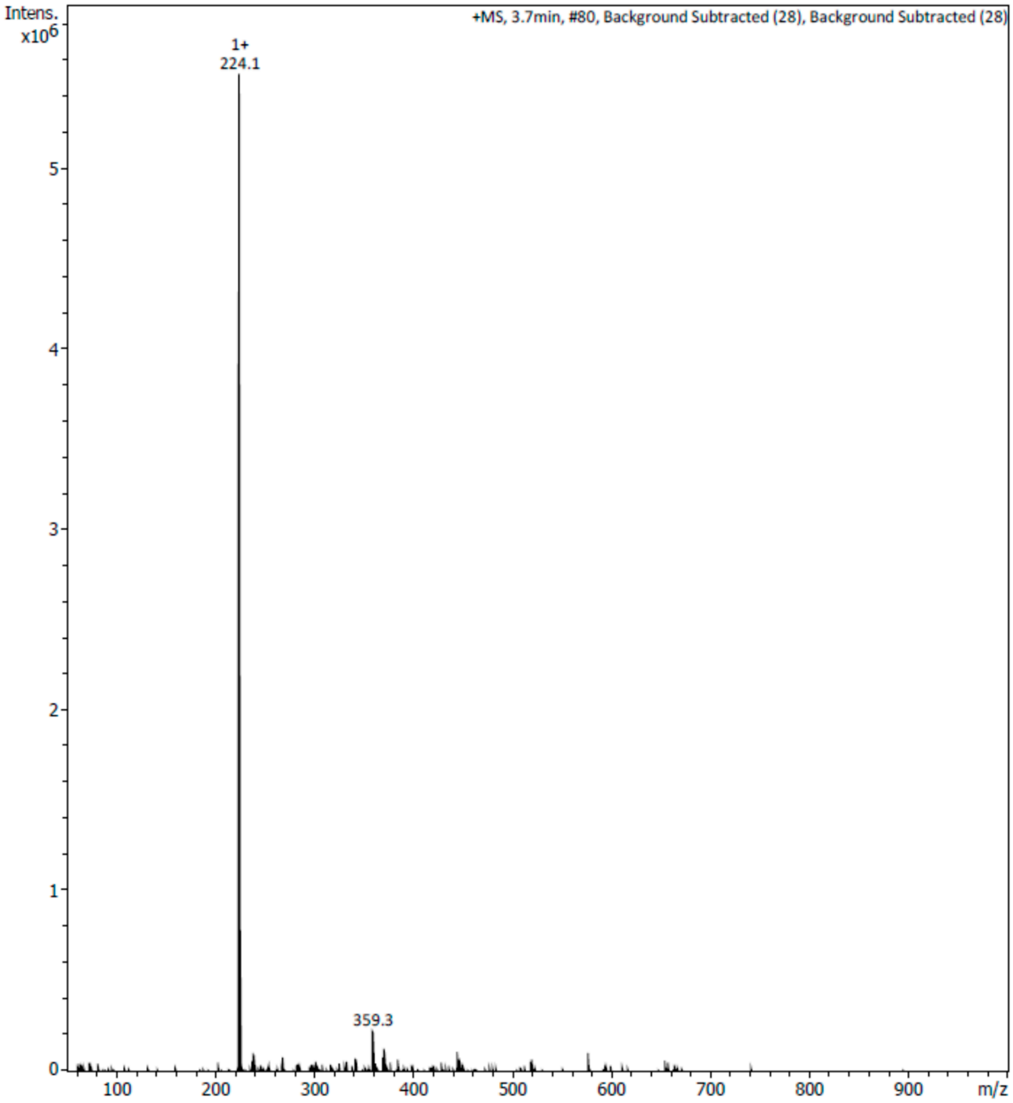

Supplement: Supplementary file 1 [file molecules-29-05058-s001.zip › molecules-3191702-supplementary.pdf]
